# MXene-Based Elastomer Mimetic Stretchable Sensors: Design, Properties, and Applications

**DOI:** 10.1007/s40820-024-01349-w

**Published:** 2024-02-27

**Authors:** Poushali Das, Parham Khoshbakht Marvi, Sayan Ganguly, Xiaowu (Shirley) Tang, Bo Wang, Seshasai Srinivasan, Amin Reza Rajabzadeh, Andreas Rosenkranz

**Affiliations:** 1 School of Biomedical Engineering, McMaster University, 1280 Main Street West, Hamilton, ON L8S 4L8 Canada; 2 Department of Chemistry and Waterloo Institute for Nanotechnology (WIN), University of Waterloo, 200 University Ave West, Waterloo, ON Canada; 3 Centre for Eye and Vision Research (CEVR), 17W Hong Kong Science Park, Shatin, Hong Kong People’s Republic of China; 4 Chair of Functional Materials, Department of Materials Science and Engineering, Saarland University, Saarbrücken, Germany; 5 W Booth School of Engineering Practice and Technology, McMaster University, 1280 Main Street West, Hamilton, ON L8S 4L7 Canada; 6 Department for Chemical Engineering, Biotechnology and Materials, University of Chile, Santiago, Chile

**Keywords:** Flexible sensor, 2D nanomaterials, MXene, Wearable and conductive, Applications

## Abstract

Flexible sensors based on MXene-polymer composites are highly prospective for next-generation wearable electronics used in human–machine interfaces. One of the motivating factors behind the progress of flexible sensors is the steady arrival of new conductive materials. MXenes, a new family of 2D nanomaterials, have been drawing attention since the last decade due to their high electronic conductivity, processability, mechanical robustness and chemical tunability. In this review, we encompass the fabrication of MXene-based polymeric nanocomposites, their structure–property relationship, and applications in the flexible sensor domain. Moreover, our discussion is not only limited to sensor design, their mechanism, and various modes of sensing platform, but also their future perspective and market throughout the world. With our article, we intend to fortify the bond between flexible matrices and MXenes thus promoting the swift advancement of flexible MXene-sensors for wearable technologies.

## Highlights

- MXenes, a new family of 2D nanomaterials, have been drawing notable attention due to their high electrical conductivity, processability, mechanical robustness and chemical tunability.
- Flexible sensors based on MXene-polymer composites are highly prospective for next-generation wearable electronics used in human–machine interfaces.
- With our article, we intend to fortify the bond between flexible matrices and MXenes thus promoting the swift advancement of flexible MXene-sensors for wearable technologies.

## Graphical abstract

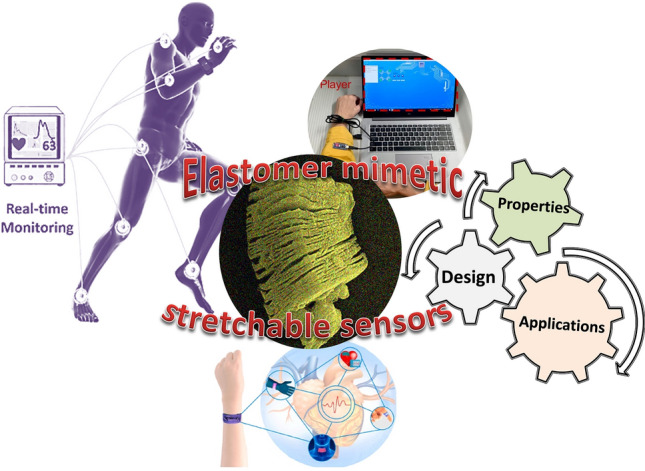

## Introduction

The notable advancement in consumer electronics, artificial intelligence, and clinical medicine has increased the demand for flexible sensors. These sensors rely heavily on sensitive materials that possess a high flexibility and an appropriate conductivity to achieve optimal performance. The increasing potential of flexible wearable and implantable sensors has boosted research efforts within academic and industrial sectors. Different types of flexible sensors including piezoresistive sensors, gas sensors, human motion recognition, facial expression recognition, signature recognition, strain sensors, among others, exhibit significant prospects for their application in sport fitness, health monitoring and evaluation, clinical diagnosis, and industrial robots [1–8]. Wearable health sensors have been developed for a variety of uses due to recent developments in sensor fabrication. Patients with limited access to medical care, as well as those in the recovery phase, can benefit from using wearable sensors [9–11]. Wearable sensors allow for a continuous monitoring of health parameters without the need for frequent hospital visits, reducing the financial burden of healthcare [12]. However, each wearable sensor has its own set of restrictions when it comes to the real-time monitoring of parameters [13].

Consequently, multiple sensors are required to measure a wide range of health indicators [14]. These sensors have been developed over time using multiple manufacturing techniques for use in healthcare, pathogen detection, and clinical situations [15]. An electrocardiogram (ECG) is a standard procedure to record the heart’s electrical activity that can provide useful information about the respective heart rate and rhythm. To get beyond the limitations of traditional dry and wet electrodes, researchers developed and connected carbon nanotube (CNT) polydimethylsiloxane (PDMS) composites to a standard ECG [16]. They used variable CNT concentrations to create a wide range of electrodes. At the same time, a sensitive wearable sensor capable of tracking the heart rate was developed. To this objective, polymeric nanofibers were employed and supported on a thin PDMS layers [17]. Both the static force of a heartbeat and the dynamic motion of a water droplet were detected by the constructed sensor, which led to the development of biocompatible sensor capable to measure the blood flow [18].

Recent advancements in the synthesis of nanomaterials and the research conducted on nanotechnology have showcased significant implications across all domains of science, engineering, and healthcare [19–24]. The flexible sensors necessitate several key characteristics, including an improved sensitivity, wide detection range, rapid reaction time, low detection limit, good linearity, and superior long-term stability [25]. While various sensors exhibit different operational methods, they have a common composition consisting of flexible substrates and active materials with good conductivity [26, 27]. Therefore, it is essential to carefully engineer and control the microstructure of the active material to effectively fulfill the aforementioned criteria. However, usual active materials employed in sensors are metals or semiconductors, which have excellent conductivity but unavoidably limit the overall sensitivity. Furthermore, due to their inherent rigidity, metals and semiconductors frequently fail to match the flexibility demand. Consequently, it is vital to identify alternative materials with superior conductivity and flexibility, as well as easy-to-control morphology, to design and assemble stretchable sensors with high sensitivity and a wide sensing range.

MXenes are an extensive family of 2D materials [12, 28–36] with an excellent electrical conductivity (up to ≈ 10^4^ S cm^−1^) [37], large specific surface area, unique layered-structure and high specific capacitance (up to ≈ 1500 F cm^−3^) [38]. They are typically synthesized from the parent MAX phases (M_n + 1_AX_n_ with “M” as early transition metals, “A” group 13–16 elements, “X” carbon and/or nitrogen, and n = 1–4). Gogotsi et al. reported the synthesis of Ti_3_C_2_T_*x*_ in 2011 by chemical wet-etching of Ti_3_AlC_2_ precursors [39]. So far, different MXenes have been successfully synthesized, including Ti_2_C, V_2_C, Nb_2_C, Ti_4_N_3_, TiNbC, Ti_3_CN, and Mo_2_TiC_2_ [29]. Ti_3_C_2_T_*x*_ has found uses in a wide range of fields, including energy storage, sensing, catalysis, antennas, and neural interfaces [30, 35, 38, 40–44]. However, there are certain limitations associated with MXenes that hinder their progress in attaining the desired levels of flexibility and durability required for wearable electronics. These downsides include insufficient mechanical properties, susceptibility to restacking, relatively small lateral dimensions, and inadequate stability in oxygen-rich environments [45, 46]. Collectively, these factors significantly impede the advancement of MXenes in various fields.

Fortunately, the unique characteristics of MXenes make it simple to combine with other materials, providing an opportunity to successfully merge the excellent capabilities of different materials in a complementary way [47]. MXene-based composites with great electrical conductivity and flexibility are thus interesting materials for stretchable electronics [48, 49]. MXene (M_n + 1_X_n_T_*x*_) flakes with high aspect ratios (up to ≈ 10^6^) and numerous surface terminations (T_*x*_) are beneficial to produce polymer composites with novel features. For instance, composite films comprised of Ti_3_C_2_T_*x*_ and sodium alginate showed a superior electromagnetic interference shielding efficiency compared to other synthetic composite materials of comparable thickness [50]. MXene-polymer composites have demonstrated enhanced mechanical properties due to the inherent flexibility of polymers, thereby rendering them suitable for a wide range of applications [51, 52]. Moreover, the use of polymers can effectively enhance the interlayer spacing and mitigate the aggregation of MXene nanosheets, promoting the overall stability of the composite structure. Additionally, polymers confer special functionalities to MXene by offering an abundance of electron–ion channels and interaction sites, which, in turn, enhances the performance of MXene-polymer composites. A domain of significant relevance, in which MXenes have a competitive edge compared to conventional fillers [53], connects with composite fibers that demand concurrent electrical conductivity and elasticity. The fundamental prerequisite for sensing physical deformations such as strain is a combination of conductivity and stretchability. Various materials have been utilised as fillers to yield composite fibres. These materials include conducting polymers like poly(3,4-ethylenedioxythiophene):poly(styrenesulfonate) (PEDOT:PSS) [54], carbon nanotubes [55], reduced graphene oxide [56–60], and silver nanoparticles [61–64], among others [53, 65–68]. When incorporated into a textile material, these fibers possess the potential to facilitate various applications such as monitoring body movements, providing sport coaching, aiding in rehabilitation, enabling remote health monitoring, and enhancing entertainment experiences [69]. To date, comprehensive reviews on MXenes have primarily concentrated on their applications in energy storage, conversion, EMI shielding, piezoresistive sensors, and photoelectric catalysis [28, 29, 70, 71]. In comparison, there are fewer reports employing MXenes-based elastomer mimetic stretchable sensors. This article presents an overview of the latest advancements in flexible MXene-based composites designed for stretchable sensors focusing on the fabrication strategies, working mechanism, and performance. Finally, the current challenges and future possibilities are also discussed.

## Fabrication Strategies and Properties of MXenes/Polymer Nanocomposites (MPCs)

MXenes have been used as 2D fillers in polymer matrices to design composite materials with versatile properties and significant potential in catalysis, sensor, energy storage, membrane, and biomedicine [72–74]. Similar to other 2D materials like graphene and hexagonal boron nitride (h-BN), MXenes provide a strong foundation for expanding a substantial pathway for making composites. The fabrication strategies including in-situ polymerization, solution mixing, or other approaches, impact not only the homogeneity of the nanocomposite but also effect the interfacial interactions between MXenes and the polymeric matrix. These interactions significantly contribute to the resulting mechanical, thermal, and electrical properties. MPCs can be generated by various polymer fabrication and forming techniques, including in-situ polymerization and ex-situ blending. In this section, we will focus on MXene-based thermoplastic and thermosetting polymers.

**Fig. 1:**
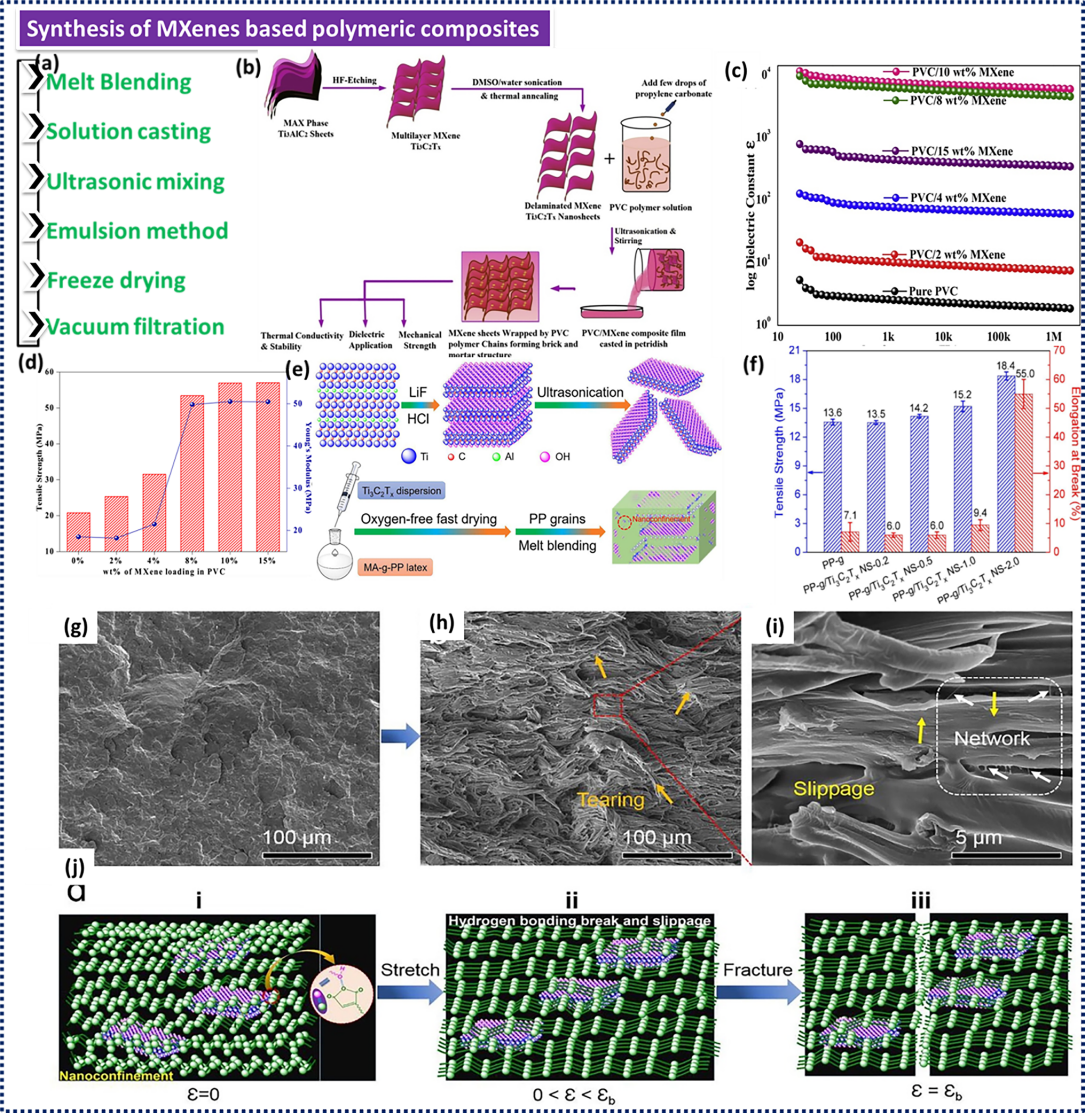
**a** Different techniques for synthesising MPCs. **b** Schematic illustration showing the preparation of d-Ti_3_C_2_T_*x*_ (HF-etching prior to delamination by DMSO and water and followed by high-temperature annealing) and PVC/Ti_3_C_2_T_*x*_ nanocomposite films. **c** Dielectric constant and (**d**) Young’s modulus as well as tensile strength of PVC/MXene nanocomposites. Reproduced with the permission from Ref. [90], Copyright Elsevier 2020.** e** Schematic representation of melt blending for the fabrication of Ti_3_C_2_T_*x*_/PP nanocomposites. **f** Tensile strength and elongation at break of the Ti_3_C_2_T_*x*_/PP nanocomposites as a function of the Ti_3_C_2_T_*x*_ content. SEM images of 2.0 wt% Ti_3_C_2_T_*x*_/PP nanocomposite as well as **g** their fracture surface after freeze-brittle fracture. **h** and **i** Stretch-fracture surface; **j** Schematic models of mechanical failure (including H bonding break and slippage) of PP-g/Ti_3_C_2_T_x_ NS-2.0. Reproduced with the permission from Ref. [91], Copyright Elsevier 2019

The most frequently used matrixes for synthesizing MXene/thermosetting (MTS) polymers are epoxy resins. In contrast, ultra-high molecular weight polyethylene (UHMWPE), polypropylene (PP), polyvinyl alcohol (PVA), polyvinyl chloride (PVC), and polyurethane (PU) have been employed for MXene/thermoplastic (MTP) polymers. The most important pathways for the synthesis of MPCs are depicted in Fig. 1a, which include ultrasonic mixing, freeze-drying, solution blending, vacuum filtration, and melt blending. In this regard, ultrasonic blending is straightforward since the polymer and MXene mixtures are first stirred. Subsequently, ultrasonication is used to achieve the stable and homogeneous MXene dispersion throughout the matrix. This technique has been mainly utilised for epoxy-based composites. Solution casting is another approach to prepare MPCs. For this method, both MXene and polar polymer components are separately dispersed in a suitable solvent. Following this, both solutions are mixed prior to solvent evaporation. Solution blending works well with polar polymers including PVA, PVC, and PU, while melt blending is a feasible route for MTP polymers such as PP. After melting the polymer, MXenes are mixed with the polymer using an extruder. Vacuum filtration and freeze–drying have been proposed for the fabrication of thin films and 3D structures using stable MXene/polymer solutions. According to the literature, the electrical, thermal, mechanical, corrosive, and tribological attributes of both thermosetting and thermoplastic polymers can be greatly improved with the addition of MXenes, thus making them suitable for a wider range of applications [75, 76]. Several MXene-based nanocomposites and their specific application areas are summarized in Table 1.

**Table 1:** Overview of typical MXenes-based polymer composites with the respective application area and key features

MXenes type	Polymer matrix	Application area	Features	References
AgNWs/Ti_3_C_2_T_x_	PU	Strain sensing	1000 cycling stability, response/recovery time of 344/344 ms	[77]
Ti_3_C_2_T_x_	Chitosan and PU	Pressure sensing	High compressive strength	[78]
Mo_2_C	PVA	Humidity sensing	1/1.8 s recovery time	[79]
TiO_2_/Ti_3_C_2_T_x_	Nafion	Peroxide sensing	Linear range of 0.1–380 μM, sensitivity of 447.3 μA (mM cm^2^)^−1^, detection limit of 14 nM, 94.6% left after 60-day storage	[80]
GO_x_/Au/Ti_3_C_2_T_x_	Nafion	Glucose sensng	Sensitivity of 4.2 μA (mM cm^2^)^−1^, detection limit of 5.9 μM (S/N = 3)	[81]
Ti_3_C_2_T_x_	PVA	Capacitor	Electrical conductivities of 2.2 × 10^4^ S m^−1^, volumetric capacitance of 530 F cm^−3^ at 2 mV s^−1^	[82]
Ti_3_C_2_–S/d-Ti_3_C_2_	PP	Battery	Reversible capacitance	[83]
Ti_3_C_2_T_x_	PDT	Supercapacitor	Capacitance of 52.4 mF cm^−2^ (3.52 F cm^−3^) at 0.1 mA cm^−2^, 10,000 charge–discharge cycles (100%)	[84]
Ti_3_C_2_T_x_	PVDF	Antibacterial	Antibacterial rate of 73 and 67% against Bacillus subtilis *Escherichia coli*, respectively	[85]
Ti_3_C_2_T_x_	PAN	Air purify	2.5 removal efficiency of 99.7%, low pressure drop of 42 Pa	[86]
Ti_3_C_2_T_x_	PANI/PP	EMI	~ 40 dB	[87]

### MXene-based Thermoplastic Polymer Composites and Their Properties

In this section, we discuss different synthesis strategies for MXenes-reinforced thermoplastic polymer composites. Liu et al. used delaminated Ti_3_C_2_T_*x*_ nano-sheets (d-Ti_3_C_2_) to prepare d-Ti_3_C_2_T_*x*_/PVA films by solution blending [88]. X-ray diffraction (XRD) and scanning electron microscopy (SEM) verified the homogeneous dispersion of d-Ti_3_C_2_ in PVA, which helped to notably improve their mechanical and thermal properties. The improved modulus of elasticity, elongation at break, and tensile strength of the d-Ti_3_C_2_/PVA films was caused by the H-bonding between the PVA matrix and d-Ti_3_C_2_. Moreover, the addition of 0.5 wt% d-Ti_3_C_2_T_*x*_ improved the crystallinity of PVA by 42%, which also contributed towards their improved mechanical properties. Pan et al. generated d-Ti_3_C_2_T_*x*_/PVA composites by casting and evaporation [89]. Incorporating d-Ti_3_C_2_T_*x*_ into PVA resulted in a slower rate of thermal decomposition and less weight loss compared to pure PVA films. The incorporation of 1 wt% of d-Ti_3_C_2_T_x_ decreased both the peak heat release rate (PHRR) and total heat release (THR) by 25%. At the same time, the MXene addition led to an increase in both tensile strength and elongation at break compared to that of a pure PVA film. Mazhar et al. used delaminated and annealed Ti_3_C_2_T_*x*_ nano-sheets to create PVC/Ti_3_C_2_T_*x*_ composites by solution casting aided by vigorous sonication (Fig. 1b) [90]. According to the percolation hypothesis, the embedded d-Ti_3_C_2_T_*x*_ in PVC supported the generation of a conducting-insulating-conducting network by enhancing the AC conductivity of the nanocomposites. Many PVC/ Ti_3_C_2_T_*x*_ interfaces at the percolation threshold of 10% MXene encourages interfacial polarisation, endowing the produced nanocomposites with significant dielectric properties as shown in Fig. 1c. The thin PVC/Ti_3_C_2_T_*x*_ films exhibited a very high flexibility, an enhanced thermal conductivity of 3.48 W mK^−1^, exceptional thermal stability (683.8 °C) and an excellent mechanical stability. Figure 1d depicts the tensile strength and tensile modulus of the PVC/Ti_3_C_2_T_*x*_ composites as a function of the MXene content. By adding 15 wt% d-Ti_3_C_2_T_*x*_, the tensile strength and modulus increased by 173% and 177%, respectively. The strong interfacial interaction between d-Ti_3_C_2_T_*x*_ and PVC was described as the primary cause for improved mechanical properties. Shi et al. produced ultra-thin Ti_3_C_2_T_*x*_/PP nanocomposites with notably improved characteristics using oxygen-free rapid drying aided solution casting and melt blending [91]. The enhancement in thermal stability and mechanical properties that result from the utilization of Ti_3_C_2_T_*x*_ surpassed those of comparable 2D nanomaterials. This was due to the integration of a nanoconfinement structure induced by H bonds with the physical barrier effect of ultra-thin Ti_3_C_2_T_*x*_ nanosheets. Figure 1e provides a schematic representation of the melt blending approach to prepare Ti_3_C_2_T_*x*_/PP nanocomposites. It is important to note that since PP is a non-polar polymer, maleic anhydride was employed as a compatibilizer for producing H bonds between Ti_3_C_2_T_*x*_ and PP. The results depicted in Fig. 1f demonstrate that the inclusion of 2.0 wt% Ti_3_C_2_T_*x*_ increased the resulting strength, ductility, and modulus by 35%, 674%, and 102%, respectively. The enhancement in the tensile strength of the PP nanocomposites was observed to be directly proportional to the MXene content, which was attributed to the hindrance of the mobility of the polymer chains within the nanoconfinement structure facilitated by H bonding. The incorporation of Ti_3_C_2_T_x_ also increased the storage modulus from 1.36 to 1.46 GPa, which was traced back to the enhanced adhesion at the Ti_3_C_2_T_*x*_/PP interface, which was supported by the presence of multiple H bonds. Figure 1g–j depicts the proposed mechanisms for the mechanical reinforcement of the nanocomposites. The application of tensile load resulted in the occurrence of shear deformation and slipping phenomena due to the high mobility of the Ti_3_C_2_T_*x*_/PP interface, leading to increased energy absorption. However, the existence of confinement at the nanoscale level diminishes the deformation of the polymer chains and the subsequent failure of samples, thereby enhancing the strength of samples. Schematic models of mechanical failure (including H bonding break and slippage) of Ti_3_C_2_T_x_/PP nanosheet (NS) 2.0 is shown in Fig. 1j. Prior to the extension test (elongation at break, ɛ = 0), the PP-g/Ti_3_C_2_T_x_ NS-2.0 had a 3D structure made from numerous H-bond interactions between the Ti_3_C_2_T_x_ NS and the PP-g. The red box indicates the formation of a Ti_3_C_2_T_x_ NS-centered nano-confinement phase due to these H-bond interactions. The phenomena of stress whitening is noticed at this stage, suggesting the presence of shear deformation when stretching is conducted (0 < ε < εb). When further increasing the external load, microcracks within the nanocomposite gradually develop into macrocracks and even cracks, thus resulting in the rupture of the PP-g chains (ε = εb). Zhi et al. verified that the incorporation of MXenes into polyurethanes (Pus) by emulsion method led to a notable enhancement in their mechanical characteristics, such as yield stress, tensile strength, and hardness, even at a low MXene content [92]. The usage of 0.5 wt% MXenes increased the resulting yield stress, tensile strength, and hardness of Pus by 70%, 20%, and 13%, respectively. These findings were traced back to the homogeneous and effective MXene dispersion in the PU matrix, which was achieved by ball milling of MXene-filled polyurethane emulsions.

**Fig. 2:**
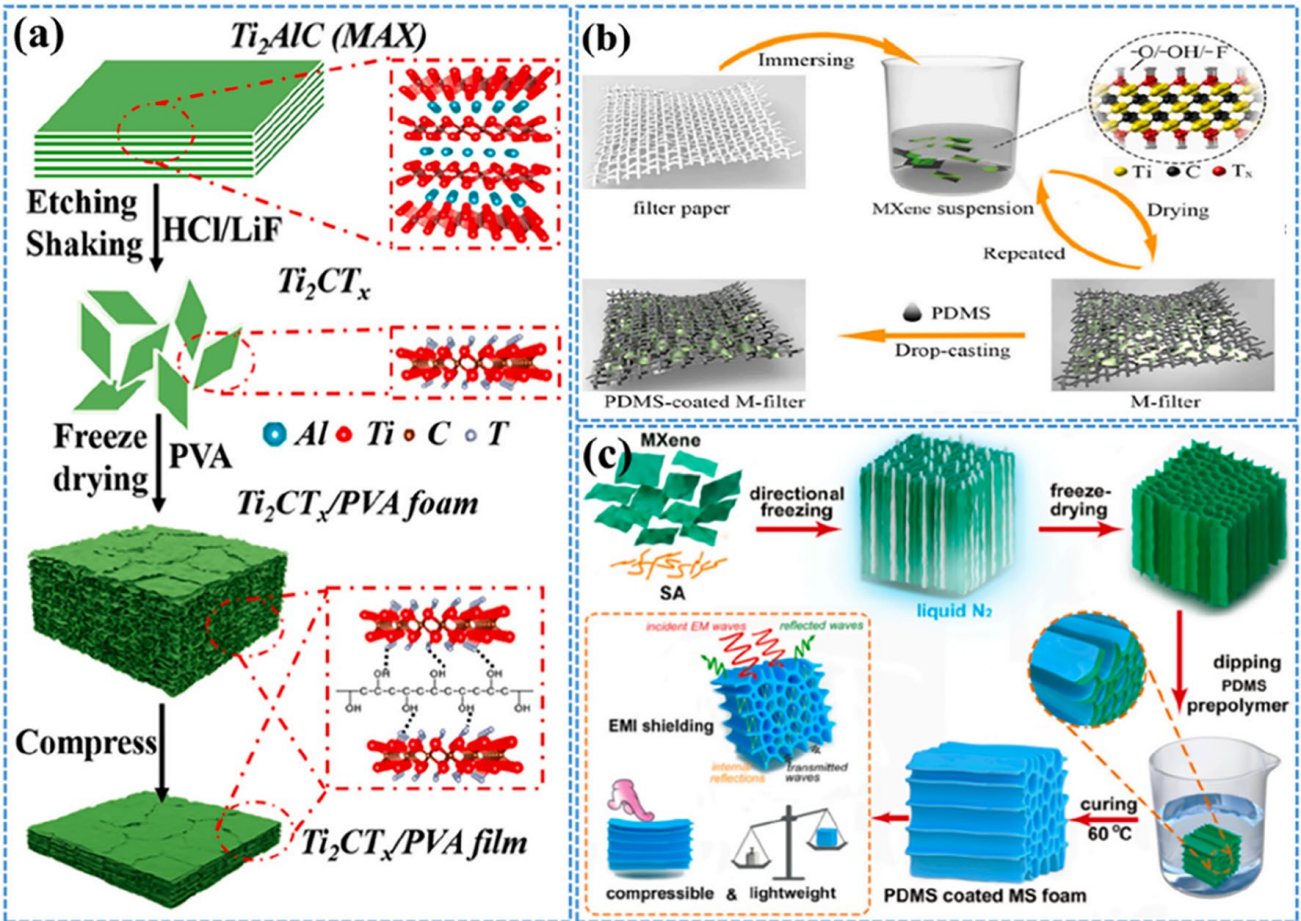
**a** Graphical representation of the fabrication of composite foams and films made from f-Ti_2_CT_*x*_/PVA. Reproduced with the permission from Ref. [93], Copyright American Chemical Society 2019. **b** Schematic representation of the synthesis procedure for freestanding nanocomposites. Reproduced with the permission from Ref. [94], Copyright Elsevier 2020. **c** Diagram depicting the generation of hybrid aerogels and PDMS-coated MS foams. Reproduced with the permission from Ref. [95], Copyright Elsevier 2020

Porous composite foams consisting of few-layer Ti_2_CT_*x*_ and PVA have been successfully fabricated by freeze-drying (Fig. 2a) [93]. They demonstrated that effective impedance matching, resulting from the utilization of various porous structures, internal reflection, and polarisation effects (including dipole and interfacial polarisation), collectively contributed to enhance the absorption efficiency and superior EMI shielding performance. MXene/cellulose nanofiber (CNF) composite papers were generated by vacuum-assisted filtration [94]. A thin layer of polydimethylsiloxane (PDMS) was subsequently introduced to safeguard MXene networks from oxidation, as depicted in Fig. 2b. Wu et al. produced compressible and electrically conductive polydimethylsiloxane (PDMS)-coated MXene foams by creating 3D MXene aerogels followed by the application of a thin PDMS layer as shown in Fig. 2c [95]. This resulted in an improved structural stability and durability of the porous architectures. The hybrid aerogel showed an exceptional conductivity of 2211 S m^−1^ due to its unidirectional porous channels. Sheng et al. fabricated Ti_3_C_2_T_*x*_ /PU nano-composites by environmentally friendly melt blending [96]. The pre-treatment of MXenes with PEG resulted in an increased d-spacing due to intercalation, which effectively prevented agglomeration and significantly enhanced the dispersion of MXene in TPU.

**Fig. 3:**
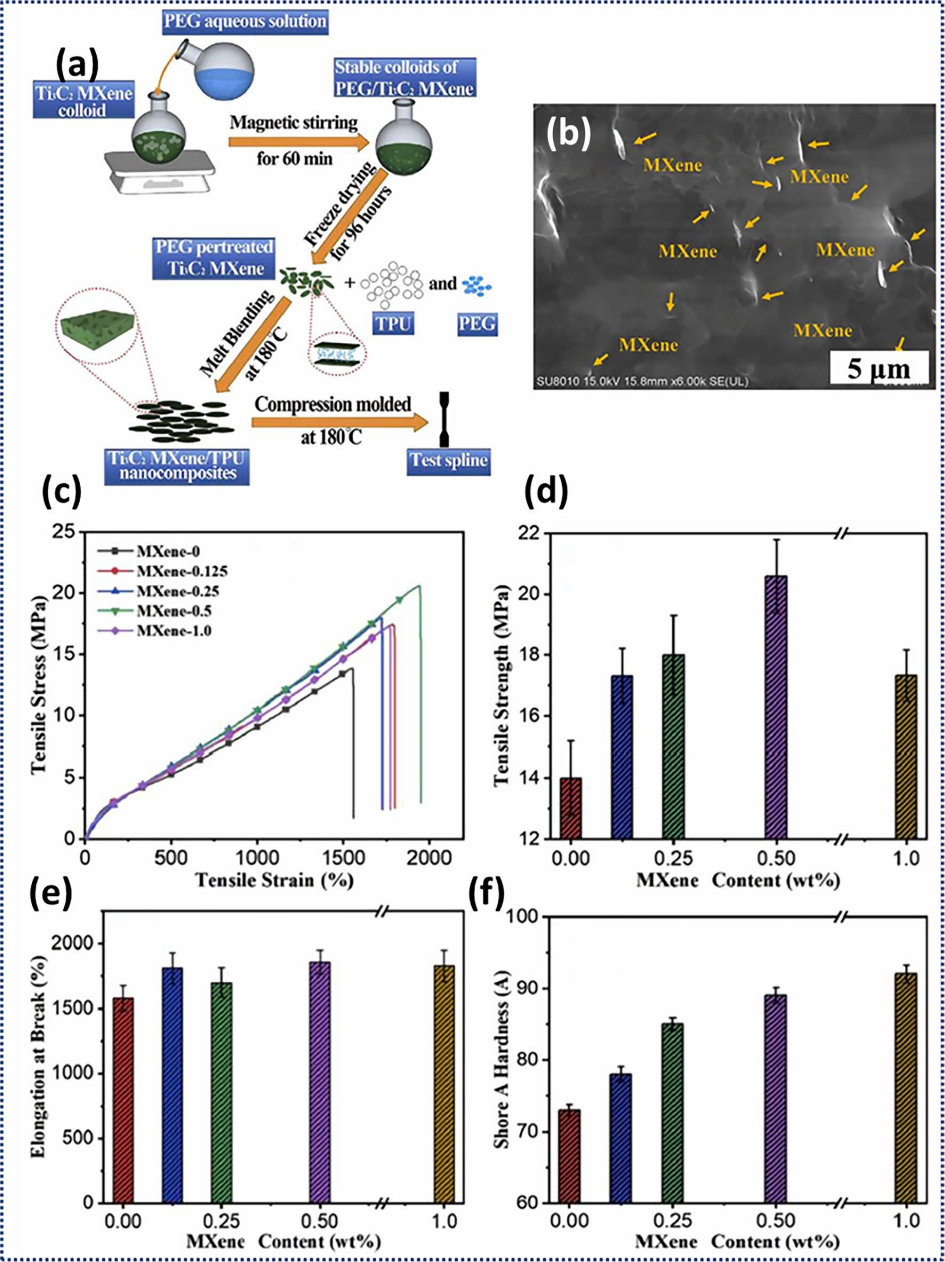
**a** Schematic representation of melt blending used to prepare Ti_3_C_2_T_x_/TPU nanocomposites with the **b** corresponding SEM image of 1.0 wt% Ti_3_C_2_T_x_/TPU. Resulting mechanical properties of the Ti_3_C_2_T_x_/TPU nanocomposites as a function of the MXene content including **c** tensile stress–strain curves, **d** tensile strength, **e** elongation at break, and **f** hardness. Reproduced with the permission from Ref. [96] Copyright Elsevier 2019

Figure 3 summarizes the fabrication, morphology, and mechanical properties of Ti_3_C_2_T_*x*_/TPU nanocomposites. The incorporation of Ti_3_C_2_T_*x*_ (0.125, 0.25, 0.5, and 1.0 wt%) increased the resulting tensile strength by 23.6%, 28.6%, 47.1%, and 25%, respectively, which the storage modulus showcased an increase of 12.5%, 24.6%, 39.8%, and 59.8%, respectively. This was attributed to an efficient load transfer mechanisms between the stiffer MXenes and the softer TPU matrix. Moreover, MXene/TPU nanocomposites demonstrated enhanced thermal properties because of the physical barrier effect of Ti_3_C_2_T_*x*_. Authors suggested that the observed improvements can be attributed to the ability of Ti_3_C_2_T_*x*_ to reduce the chain mobility and diffusion in the polymer matrix, ultimately enhancing the polymer’s thermal stability. Luo et al. used vacuum-assisted filtration to produce flexible and remarkably conductive nanocomposite films of Ti_3_C_2_T_*x*_ and natural rubber (NR) due to the creation of interconnected MXene networks within the NR matrix [97]. The electrostatic repulsion, which arises from the negative charges present in both MXenes and NR facilitated the preferred distribution of MXenes at the interfaces of NR. This resulted in the formation of a well-connected network that enabled an effective electron transport and load transfer, even at low MXene contents. The produced nanocomposites exhibited an exceptional electrical conductivity of 1400 S m^−1^ and remarkable electromagnetic interference (EMI) shielding performance of 53.6 dB for a MXene content of 6.71 vol% at a thickness of only 251 μm. The MXene network also showed beneficial effects regarding the resulting mechanical properties leading to an increase of the tensile strength and modulus by 7 and 150 times, respectively. This study presents a straightforward and expandable method for producing flexible materials with reliable EMI shielding ability and the capacity to stretch during cyclic deformations, which can be potentially used for the development of advanced flexible and foldable electronics. Table 2 summarizes the used polymer type, MXene, and fabrication methods for different MXene/thermoplastic nanocomposites.

**Table 2:** General information regarding Ti_3_C_2_T_x_ MXene/thermoplastic nanocomposites

Thermoplastic Polymer	Method of preparation	References
PU	Emulsion method	[92]
PVA	Casting/evaporation method	[89]
UHMWPE	Melt blending	[98]
PEDOT:PSS	Vacuum-assisted filtration process	[99]
NR	Vacuum-assisted filtration process	[97]
PVA	Solution blending method	[88]
PVC	Solution blending method	[90]

### MXene-Based Thermosetting Polymer Composites and Their Properties

MXenes are a good choice to enhance the mechanical and tribological performance of thermosetting polymers due to their layered architecture and tunable surface chemistry. ERs with exceptional mechanical properties, minimal shrinkage during curing, low residual stresses as well as excellent thermal and chemical resistance, are a prevalent form of thermosetting polymers that possess a broad range of applications [100–103]. The significance of improving the mechanical and tribological performance of ERs lies in their widespread use, including but not limited to polymeric gears, bearings, and coatings [104–106]. The outstanding features of thermosetting polymers make them a great fit for sensor applications. As a result of their extraordinary resistance to high temperatures and chemical inertness, these polymers are well-suited in sensors deployed in harsh situations, such as those found in automotive, aerospace, and other industrial sectors [107]. They excel an excellent stability, ensuring that the sensor’s geometry remains stable, which is a vital feature for precision and accuracy. Due to their robust mechanical properties, thermosetting polymers are an excellent choice for sensors that are subjected to mechanical loads or pressure [59]. They are ideally suited for sensors that require electrical isolation because to their high levels of electrical insulation [67, 108]. Furthermore, they can be formed into intricate geometries, facilitating unique sensor designs with enhanced functionality and seamless integration [109]. Their durability and resilience to the effects of time also ensure that sensors retain their function and sensitivity for extended periods of times. Under sustained stresses, their minimal creep reduces the potential bending to a minimum extent, while keeping their calibration and accuracy intact. While thermosetting polymers are advantageous for many sensor applications, this is not the case for all applications, which requires the investigations of alternative polymer types.

**Fig. 4:**
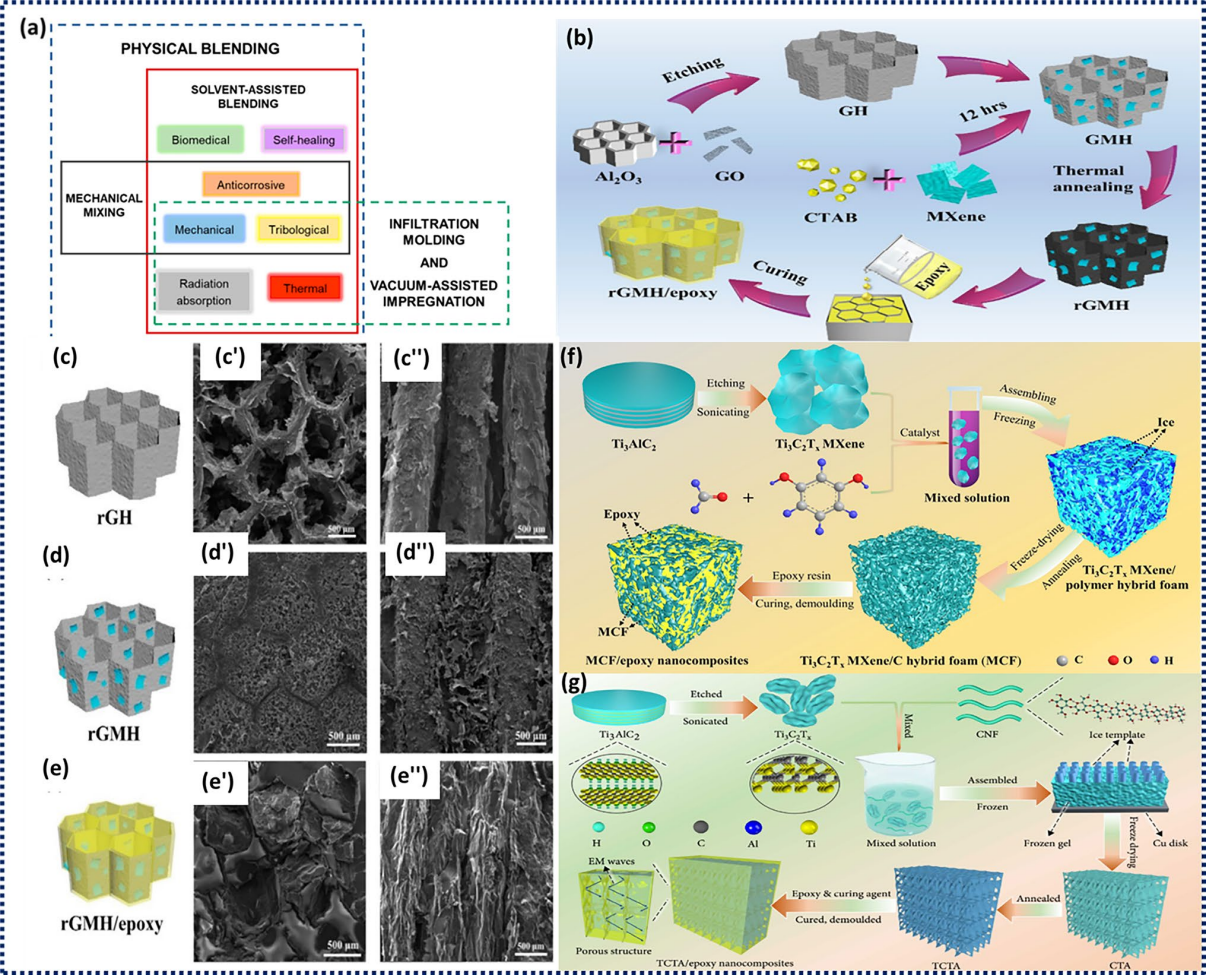
**a** Schematics of MXene/epoxy nanocomposites processing techniques. **b** Schematic illustration of the fabrication process of **c** rGH, **d** rGMH, and **e** rGMH/epoxy nanocomposites. Axial and radial SEM images of **c'**, **c"** rGH, **d'**, **d"** rGMH, and **e'**, **e"** rGMH/epoxy nanocomposites, respectively. Reproduced with the permission from Ref. [111], Copyright Elsevier 2020. **f** Schematic representation of the fabrication process employed to produce MCF/epoxy nanocomposites. Reproduced with the permission from Ref. [112], Copyright Elsevier 2019. **g** Graphical representation of the synthesis method for TCTA/epoxy nanocomposites. Reproduced with the permission from Ref. [113] Copyright American Association for the Advancement of Science 2020

The approach to manufacture ER-MXene nanocomposites is crucial for the resulting composite characteristics. Appropriate ways to fabricate these composites must consider the adaptability to incorporate fillers, ease of use, and ability to achieve uniform filler dispersion within the polymeric matrix. Figure 4a depicts the target attributes of ER-MXene nanocomposites. Zhang et al. verified the self-lubricating and anti-friction properties, along with high toughness and low creep strain, for Ti_2_CT_*x*_/ER nanocomposites with variable MXene content [110]. The findings indicate that the Ti_2_CT_*x*_ nano-sheets were intercalated and exfoliated by the EP molecular chains, which led to their intertwining, thus increasing the interfacial area between the inorganic additive and polymer matrix. The incorporation of appropriate mass fractions of Ti_2_CT_*x*_ enhanced the fracture toughness and flexural strength of EP composites to 17.8 kJ m^−2^ and 98 MPa, respectively, which represent a significant increase of 76% and 66%, respectively. Song et al. reported the construction of honeycomb structural rGO (rGH) utilizing Al_2_O_3_ honeycomb plates as templates. Subsequently, a solution of MXene was blended with hexadecyl trimethyl ammonium bromide (CTAB) to induce negative surface charges on their surface [111]. Figure 4b displays the schematic illustration for the generation of rGMH/epoxy nanocomposites. The successful synthesis of rGMH exhibited an exceptional electrical conductivity and high load-bearing capacity. The synthesis process involves the electrostatic adsorption of MXene onto rGH through self-assembly. After curing at 120 °C for 5 h, the epoxy resin and curing agent blends were injected into the aforesaid rGMH to produce the rGMH/epoxy nanocomposites. The SEM images of rGH, MXene, and rGMH/epoxy nanocomposites in radial and axial directions are presented in Fig. 4c'–e''. The uniform stacking of rGO into rGH can be facilitated through the use of an Al_2_O_3_ honeycomb. This results in a surface of rGH that exhibits dense and continuous folded sheet structures. Upon complete filling of the hollow cells of rGH, MXene adheres closely to the surface of rGH and establishes interconnections to generate a continuous three-dimensional framework of rGH-MXene-rGH. Epoxy resin primarily covers spaces between honeycombs and holes between MXene sheets while preserving the original honeycomb architecture. The findings of the study revealed that the electrical conductivity (σ) of pure epoxy was measured at 2 × 10^−10^ S m^−1^. However, upon the inclusion of 1.2 wt% rGMH, the σ value of the rGMH/epoxy nanocomposites significantly increased to 43.5 S m^−1^. The incorporation of 1.2 wt% rGMH results in an increase in EMI SE values from 2 to 36 dB. Further, the incorporation of MXene into the rGMH/epoxy nanocomposites resulted in enhanced σ values and EMI SE values. The optimal values for σ (387.1 S m^−1^) and EMI SE (55 dB) were observed in rGMH/epoxy nanocomposites containing 3.3 wt% MXene. Lei et al. reported the preparation of few-layered Ti_3_C_2_T_x_ MXene through the utilization of ionic intercalation and sonication-assisted technique [112]. A hybrid foam (MCF) consisting of porous 3D Ti_3_C_2_T_x_ MXene/C was produced through a sol–gel process and subsequent thermal reduction. To make hybrid gel using the sol–gel method, Ti_3_C_2_T_x_ MXene was combined with a solution containing resorcinol and formaldehyde. After that, MCF was formed by freeze-drying and then being subjected to heat reduction. The MCF/epoxy electromagnetic interference (EMI) shielding nanocomposites were ultimately synthesised through a curing process subsequent to vacuum-assisted impregnation (Fig. 4f).

The study thoroughly examined and discussed the impact of the mass fraction of Ti_3_C_2_T_*x*_ on the electrical conductivities, and mechanical characteristics of MCF/epoxy EMI shielding nanocomposites. Wang et al. developed aligned porous architectures of 3D cellulose nanofibers (CNF)/Ti_3_C_2_T_*x*_ aerogels (CTA) with high electrical conductivity. Figure 4g depicts the schematic diagram for fabricating thermally annealed CTA (TCTA)/epoxy nanocomposites. The Ti_3_C_2_T_x_ nanosheets were exfoliated using the ion intercalation method and combined with CNF by directional freezing and freeze-drying methods. Subsequently, annealing was performed to reduce the density and improve σ. Finally, the TCTA/epoxy nanocomposites were synthesized via epoxy resin impregnation as shown in Fig. 4g [113]. At 1.38 vol% and a thickness of 3 mm, the MXene/CNF/epoxy composites exhibited an electrical conductivity (σ), EMI SE, and SE divided by thickness (SE/d) of 1672 S m^−1^, 74 dB, and 37 dB mm^−1^, respectively.

Lei et al. used ionic intercalation and sonication-assisted technique to achieve few-layered Ti_3_C_2_T_*x*_ with high electrical conductivity. Thermal reduction at medium low temperature followed by annealing helped to partially remove the existing surface terminations before producing Ti_3_C_2_T_x_/epoxy EMI shielding nanocomposites by solution casting [114]. The incorporation of Ti_3_C_2_T_*x*_ resulted in enhanced electrical conductivities of the Ti_3_C_2_T_*x*_/epoxy nanocomposites leading to a conductivity of 38 S m^−1^ for a mass fraction of 15 wt%. The nanocomposite containing 15 wt% of annealed Ti_3_C_2_T_x_ was determined to be the optimal sample, with respective electrical conductivity and EMI SE values of 105 S m^−1^ and 41 dB. The authors also verified that the incorporation of Ti_3_C_2_T_*x*_ at lower concentrations did not facilitate the construction of a conductive network, resulting in a minimal enhancement of the electrical conductivity. The characteristics of epoxy composites reinforced with high contents of Ti_3_CN fabricated by solvent processing and subsequent curing with an amine-based hardener were investigated by Hatter et al. [73]. In this regard, TEM was used to examine the degree of epoxy intercalation between MXene flakes, which helped to avoid restacking thus improving the respective dispersion. As the MXene content increased to 90 wt%, the composites demonstrated an improved mechanical performance, achieving the maximum modulus improvement of 12.8 GPa, approximately triple of that of pure epoxy.

## Microstructure and Morphology of MPCs

**Fig. 5:**
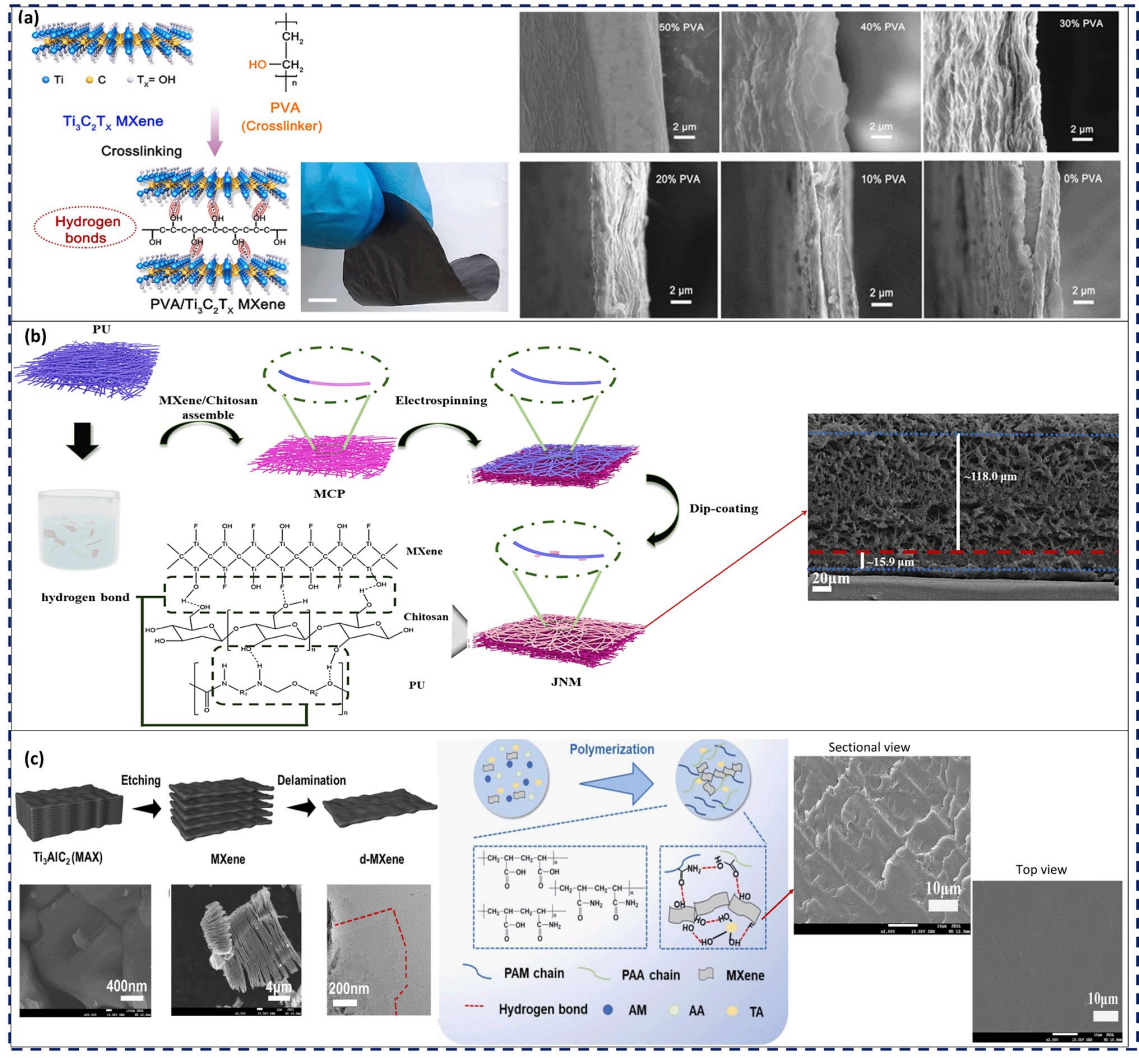
**a** Schematic depiction of the PVA/Ti_3_C_2_T_x_ MPC thin-film synthesis process, the related optical image of the resultant MPC, and the cross-sectional SEM images of the film with different content of PVA varying from 50 to 0%. Reproduced with the permission from Ref. [117], Copyright Elsevier 2021. **b** Schematic illustration of the Janus nanofibrous MXene/chitosan/polyurethane MPC with interfacial hydrogen bonding and the pertinent SEM images. Reproduced with the permission from Ref. [118], Copyright Elsevier 2023. **c** Schematic diagram of Ti_3_C_2_T_x_ nanosheet fabrication and SEM images of Ti_3_AlC_2_ and multi-layered MXene nanosheets, as well as TEM image of monolayered MXene nanosheet plus the schematic diagram of PAM/PAA/MXene/TA hydrogel fabrication and the sectional and top-view SEM images of the resultant MPC. Reproduced with the permission from Ref. [119], Copyright Elsevier 2022

MXenes and MPCs have been extensively investigated due to their unique structural and morphological characteristics. Analyzing the microstructure and morphology of MPCs is essential to understand how different fabrication approaches impact MXenes’ dispersion, interfacial interactions, and their overall structural integrity of the nanocomposite. The incorporation of polymeric compounds is proved to enhance the available specific surface area (SSA) and porosity. For instance, Li et al. [115] prepared porous Ti_3_C_2_T_*x*_/polydopamine heterostructures possessing embedded in-plane cylindrical meso-channels, where both h-bonds and electrostatic interactions directed the self-assembly of the polymeric material into MXenes’ interlayers. The SSA value of pure Ti_3_C_2_T_*x*_was found to be 21.3 m^2^ g^−1^, whereas the MPC exhibited a considerably enhanced SSA of 89.5 m^2^ g^−1^ with the average diameter of 7.6 nm. In a similar vein, the amalgamation of polyaniline (PANI) nanoparticles and single-walled carbon nanotubes (SWCNT) within Ti_3_C_2_T_*x*_, which were then assembled on the platinum electrodes, yielded flexible, porous, and electroactive film electrodes with enhanced SSA [116]. While the bare platinum and SWCNT/Ti_3_C_2_T_*x*_ film electrodes showed electrochemically active surface area (ESA) of 8.69 and 9.74 cm^2^ per 1 cm^2^ of the electrode, respectively, the ternary composite demonstrated an increased value of 10.80 cm^2^. As reported in [117], the addition of polyvinyl alcohol (PVA) as a crosslinker agent to Ti_3_C_2_T_*x*_ resulted in remarkably stable MPCs. The –OH moieties in PVA were engaged in reactive interaction with –OH-terminated MXenes, thereby initiating the formation of robust hydrogen bonding network and establishing a Ti–O–C covalent linkage. Furthermore, a trade-off relationship was observed between the amount of PVA employed and the formation of desirable porous MPCs, whereby the incorporation of 30% PVA/MXene yielded open porous lamellar structures. Conversely, higher and lower percentages of PVA saturated the interlayer voids and inhibited the uniform pore formation (Fig. 5a). A recent study proposed core–shell Janus MPCs, for which MXene/chitosan are assembled onto the polyurethane (PU) nanofibers through ultrasonication and hydrogen bonding, which is then used to collect a hydrophobic PU nanofiber membrane layer with a small thickness (Fig. 5b) [118]. This two-step synthesis resulted in the formation of a thick hydrophilic core and a thin hydrophobic shell, which allowed for minimal impact on the sensing performance due to changes in wettability. Additionally, the well-maintained porosity of the nanofibrous MPCs ensured an efficient transfer of target molecules to the hydrophilic core.

Non-porous MPCs are frequently reported when polymeric chains encapsulate MXenes through direct mixing, leading to the formation of homogeneous films. For instance, Qin et al. [119] introduced a novel MPC comprising Ti_3_C_2_T_*x*_, polyacrylamide (PAM), polyacrylic acid (PAA), and tannic acid (TA), which were held together by copious intermolecular hydrogen bonds. As portrayed in Fig. 5c, the original accordion-like MXenes were transformed into uniformly mixed MPCs, resulting in a smooth and dense microstructure. In comparison with the PAA/PAM hydrogel, the PAA/PAM/MXene/TA hydrogel displayed substantial enhancement in tensile strength, toughness, and elongation at break by 133%, 394%, and 174%, respectively. These notable improvements were attributed to the incorporation of MXenes and TA, which induced abundant intermolecular hydrogen bonds within the hydrogel network. Table 3 represents some recently proposed MPCs with reported morphological traits.

**Table 3:** Structural properties of MPCs (D_pore_: Pore Size, SSA: specific surface area, ESA: electrochemical surface area)

MPC composition	Morphology of the microstructure	References
Ti_3_C_2_T_*x*_/polydopamine	Cylindrical mesochannels	[115]
	(D_pore_ = 7.6 nm, SSA = 89.5 m^2^ g^−1^)	
Ti_3_C_2_T_*x*_/SWCNT/PANI	Hierarchical structure	[116]
	(ESA = 10.80 cm^2^ per unit area of the platinum electrode)	
Ti_3_C_2_T_*x*_/PVA	Hierarchical structure	[117]
	(D_pore_ = 0–2 µm)	
Ti_3_C_2_T_*x*_/polypyrrole	Cylindrical to spherical mesostructures and spherical macrostructure	[120]
	(D_pore_ = 7.8–52 nm, SSA = 129–188 m^2^ g^−1^)	
Ti_3_C_2_T_*x*_/cellulose acetate/sodium alginate	Unique ordered porous network	[121]
	(D_pore_ = 30–50 μm)	
Ti_3_C_2_T_*x*_/gelatin/polyacrylamide	Compact honeycomb-like porous structure	[122]
Ti_3_C_2_T_*x*_/polyacrylamide/polyacrylic acid/tannic acid	Smooth microstructure	[119]
Ti_3_C_2_T_*x*_/chitosan/polyurethane	Janus core–shell porous network	[118]
	(D_pore_ = few micrometers)	

## Structure–Property Relationship of MPCs

**Fig. 6:**
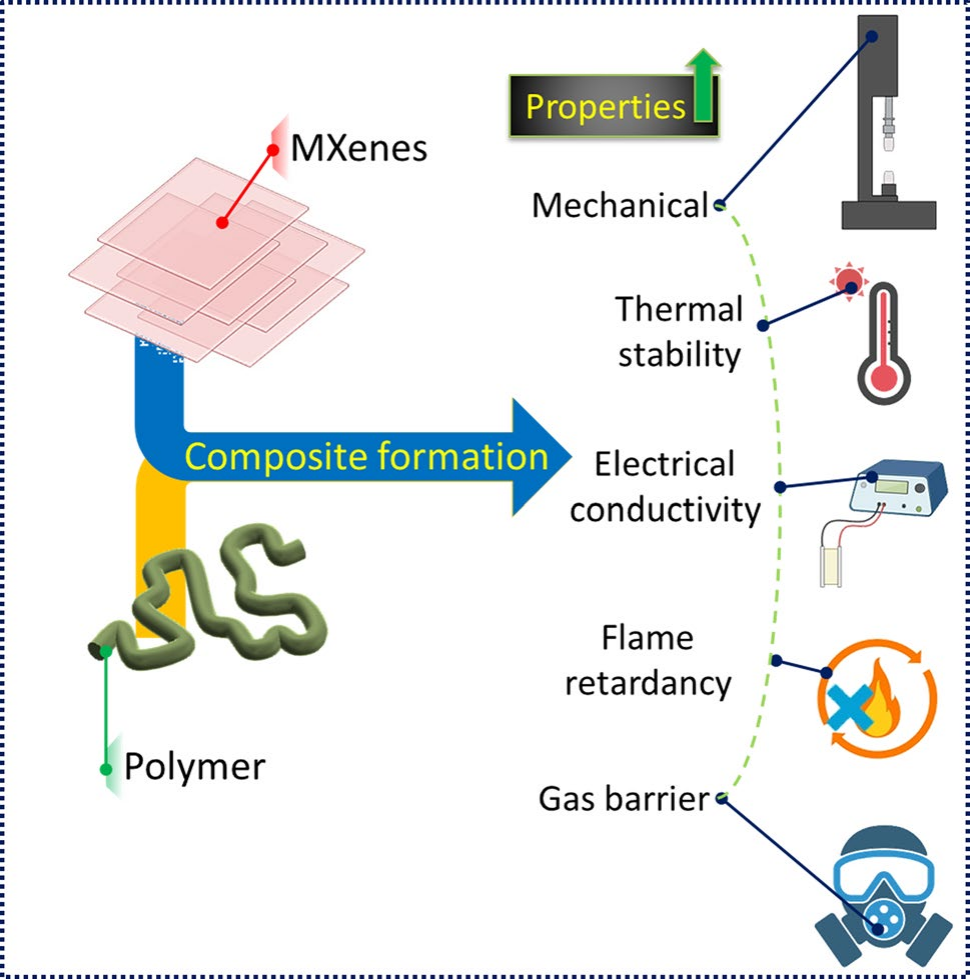
Graphical representation of the property improvements in MXenes-polymer nanocomposites in comparison to bare polymers

The connection between fabrication strategies, microstructure, morphology, and the structure–property relationship of MXenes/polymer nanocomposites is essential when exploring their applications in stretchable sensors. Polymer composites can benefit from MXenes because of their unique 2D layered structure that gives them increased strength, stiffness, toughness, and ductility (Fig. 6). Moreover, MXenes can help to increase the interfacial contact and, thus, adhesion between the MXenes and the matrix. Additionally, MXenes can be used to enhance the toughness of polymer composites due to their ability to absorb and release energy during deformation. Increasing MXenes’ content in the polymer can boost the resulting strength and stiffness, but compromise the ductility. To achieve appropriate mechanical characteristics, it is essential to properly select the type and amount of MXenes introduced to the polymer matrix. For instance, hot-pressed Ti_3_C_2_T_*x*_/UHMWPE composites demonstrated a superior hardness, yield strength, creep resistance and an improved frictional performance [123].

MXenes help to enhance the electrical conductivity of the composite. When MXenes are mixed with polymers, the resulting mixture takes on the electrical conductivity of the MXene component [124]. The polymer matrix is a supporting material that can give the composite flexibility, processability, and other desirable qualities. The conductivity of the MXene-polymer composite can be improved by optimising the makeup and processing conditions of the composite. For instance, adding more conductive materials like carbon nanotubes or graphene can make the composite more conductive, while changing the processing temperature and pressure can change the texture of the composite and make it more conductive [125].

The two-dimensional nature of MXenes allows for a better heat dissipation and improved thermal conductivity within the nanocomposite. This can make the nanocomposite suitable for high-temperature applications such as aerospace, automotive and electronic industries. Normally, polymeric systems are heat insulators, but the addition of MXenes homogeneously dispersed in the polymeric matrix can induce sufficient heat dissipation. Pan et al. reported polyvinyl alcohol and MXenes based physical mix for nanocomposite formation [89]. As per the thermogravimetric analysis (TGA) it was evident that MXenes content increment has a direct relationship for improving the thermal stability of polymer nanocomposites. Similar kind of formulation was also reported in another article where MXenes/PVA nanocomposites were thermally tested against oxygen environment [126]. They confirmed that in presence of oxygen the thermal stability of MXenes are quite better than other conventional 2D nanoparticles.

MXene/polymer composites showed a fire retardant behavior, which was connected to MXenes’ overall properties and the properties they induce in the composite When the MXene-containing composite is exposed to a flame, it forms a protective layer on the surface [127]. This layer works as a barrier for heat and gases, stopping the flame from spreading. This protective layer, based on the oxidation of MXene, causes gases like CO_2_ and H_2_O to be released, which act as a physical barrier to the flame [128]. The flame retardancy of the MXene/polymer mixture is also affected by the polymer matrix. When polymers are heated by a flame, they can start to decompose, which can release dangerous gases. Adding MXenes to the polymer matrix can make the polymer more stable at high temperatures, and reduce the gas release and help to form a protective layer that stops the flame from spreading.

MXenes have a stacked structure with a high aspect ratio, which makes them an excellent choice to create winding paths for gas molecules thus improving their ability to keep gas out. MXenes also have a large surface area and a high density of surface functional groups, such as –OH and –F, that can interact with gas molecules through hydrogen bonds and van der Waals forces, leading to better gas barrier properties [129]. Also, adding MXenes to the polymer matrix can improve the crystallinity and orientation of the polymer chains. This reduces the open volume of the polymer matrix and makes it denser, which makes it a better gas barrier [130]. MXene/polymer composites can have even better gas barrier qualities if the composition and processing conditions of the composite are made to work best. For example, raising the amount of MXenes in the composite can make it better at blocking gases, while changing the processing conditions can change the microstructure of the composite and make it even better at blocking gases [131]. Overall, the unique qualities of MXenes and the way they interact with the polymer matrix can lead to the creation of high-performance gas barrier materials that could be used in packaging, electronics, and other fields to avoid gas leakage problem.

MXene nanosheets have commendable mechanical qualities, such as a remarkable flexibility and strength, due to the presence of the robust MX bond, which is well recognised as one of the strongest chemical bonds. Kurtoglu et al. verified that the modulus of MXene was comparatively lower than that of graphene, as theoretically determined by first-principles simulations [132]. Fu et al. confirmed that MXenes’ mechanical properties surpass those of the majority of two-dimensional (2D) materials. Lei et al. have provided a detailed summary of how MXenes’ surface terminations can effectively decrease their elastic modulus and enhance their flexibility. Hence, it can be inferred that MXenes exhibit a more equitable distribution of mechanical properties compared to graphene, rendering them more appropriate for the development of flexible wearable devices [133]. The exceptional mechanical characteristics of MXene can be attributed to the presence of robust M–N or M–C bonds [47]. In comparison to graphene, MXenes have improved bend stiffness, while the monolayer of Ti_3_C_2_T_x_ demonstrated a notable Young’s modulus of about 333 ± 30 GPa. The magnetic characteristics of MXenes have been shown to be favourable due to the presence of large Fermi energy levels, which contribute to magnetic instability [134]. In addition, MXenes have demonstrated notable characteristics such as an elevated thermal conductivity, a substantial absorptivity, a pronounced hydrophilicity, and a decreased thermal expansion [135]. These remarkable characteristics render 2D MXenes suitable as nanofillers for the development of polymer composites with a superior electrical conductivity, thermal conductivity, mechanical strength, flame retardancy, among other relevant features [136].

The generation of a significant number of active terminations during etching can effectively boost the interaction between the polymer chains and the MXene flakes. This, in turn, allows for the tuning of the intralayer spacing, thus creating favourable conditions for the creation of MXene-based polymer composites with an outstanding durability [49]. Consequently, the etching procedure results in tunable functional groups and layer spacing, which, when paired with the 2D morphological structures of MXenes, facilitate the integration of various modifiers thus boosting the characteristics of the resulting MXene/polymer composites [137]. The implementation of functional modifications on MXene presents a viable solution to the aforementioned challenges. This approach not only enhances the dispersibility and compatibility of MXene with the polymeric matrices, but also enables the development of high-performance polymer composites.

## Advanced Polymer-Supported MXenes for Stretchable Sensors

Polymer-supported MXenes play a vital role in the development of stretchable sensors due to their unique combination of properties, including flexibility, electrical conductivity, and mechanical durability. Stretchable sensors based on MXenes and supported by polymers are a good fit to be incorporated into wearable electronics. These sensors enable the development of intelligent textiles and health monitoring systems that can withstand the dynamic movements of the human body.

### Synthetic Soft Matrix/MXenes-Based Stretchable Sensors

Flexible and stretchable sensors based on soft matrices and MXenes are an exciting new field in sensor technology. When MXenes are incorporated into a soft polymer, the resulting composite becomes an electrically conductive, flexible material with many potential uses. Soft and flexible polymers like PDMS are often employed in the production of synthetic soft matrix/MXenes-based stretchable sensors. These sensors can adapt to non-flat surfaces and sustain bending without breaking due to the use of a soft polymer matrix that provides the essential flexibility and stretchability. Polymer matrices combined with MXenes create composite material with excellent electrical conductivity and remarkable elasticity. The electrical conductivity of the MXenes enables the sensor to detect changes in shape or position, while the adaptability of the polymer matrix ensures that the sensor may be shaped to fit uniquely shaped objects. These types of sensors are well-suited for applications that require a stretchable and flexible sensor, such as wearable electronics, biomedical devices, and soft robots. Developing extensible sensors based on synthetic soft matrices or MXenes is an interesting and promising field of study.

Recent years have seen a rise in interest in flexible high-performance piezoresistive pressure sensors due to their promising use in smart robotics, wearable electronics, and electronic skin [138–143]. A novel method was described for producing a highly sensitive and versatile piezoresistive pressure sensor comprised of two layers [144]. It was designed on building a rough surface with micro-protrusions on polydimethylsiloxane film using sandpaper as a template and using MXenes to create electrically conductive channels. The remarkable sensitivity of 2.6 kPa over a linear pressure range of 0–30 kPa was attributed to MXenes' superior electrical conductivity and the designed micro-protrusion structure.

**Fig. 7:**
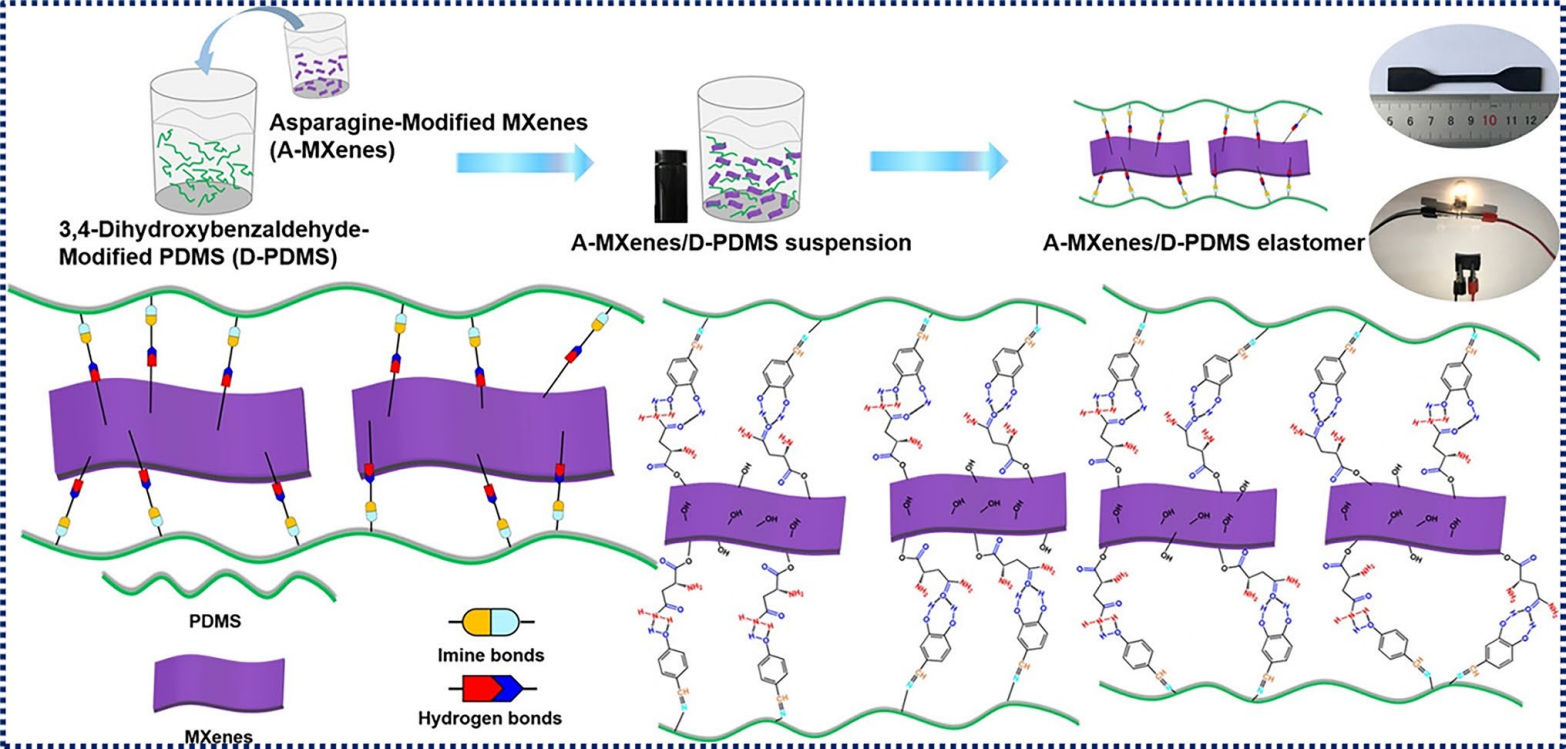
Primary steps in making MXenes/PDMS-based elastomeric nanocomposites and their physical interactions. Reproduced with the permission from Ref. [145], Copyright American Chemical Society 2020

Zhang et al. produced a conductive composite based on silicone polymer using an esterification process and a Schiff base reaction (Fig. 7) [145]. The modified MXenes were evenly distributed, contributing to the composite’s high electrical conductivity. The mechanical strength of the conductive composite containing 10 wt% A-MXenes reached 1.81 MPa with an elongation at break of 81%. The tensile characteristics and electrical conductivity could be restored to 98.4% and 97.6%, respectively, after being repaired.

Alkali-treated, puckered MXenes have been combined with micro-structured PDMS to create flexible, exceptionally sensitive piezoresistive sensors with a large detecting range [146]. To elucidate the geometric design for the ultra-wide pressure range (0–800 kPa), PDMS sheets of varying roughness were combined with as-synthesized MXenes as conducting material. These sensors have a wide range of applications in health monitoring systems and human–machine interaction due to its low detection limit (~ 17 Pa), fast response time (100 ms), good cycle stability (3000 cycles, 300 kPa), and ability to detect over a wide pressure range.

Good health requires daily health monitoring for early disease detection. Innovative health data monitoring and analysis technologies have been developed using artificial intelligence (AI). AI can help in fabricating electronic skin (e-skin) for personalized health management [147]. E-skin, also known as synthetic skin, is a thin electrical material fabricated by fusing electronic components to elastic, pliable substrates to mimic the potentials of human skin [148]. E-skin is a cutting-edge technology primarily utilized for medical purposes, such as detecting and perceiving internal life sign activities and their unprecedented monitoring. The flexible pressure sensor is one of the most essential components of robotics, health monitoring, and e-skin applications [149].

To create a self-powered tactile sensor, a flexible PDMS/MXene composite film was created via ultraviolet ozone (UVO) irradiation [150]. When compared to other battery-free tactile sensors, its 0.18 V Pa^−1^ sensitivity ranges from 10 to 80 Pa and 0.06 V Pa^−1^ from 80 to 800 Pa. This disclosed tactile sensor has potential uses for tracking complex human physiological signals and mimicking human touch feeling due to its great sensitivity for diverse pressures ranging from 10 to 800 Pa.

It is still challenging to develop a strain sensor with a high gauge factor (GF) and a wide operational range. Xu et al. made a multi-purpose strain gauge by putting carbon nanotubes (CNT) and MXenes on a porous polydimethylsiloxane (PDMS) sponge [151]. The resulting PDMS/CNT@MXene sensor demonstrated a broad sensing range (105% strain), a high degree of sensitivity (GF = 1939), quick reaction time (response/recovery time of 158/160 ms), and sufficient reliability and stability under repetitive stretching, compression, and bending cycles. The sensor may be used for medical diagnostics and a wide range of other purposes, including the detection of handwriting, heart rate, phonation, and joint movement.

The versatility of hydrogels has recently made them a hot topic because of their possible uses in fields including soft electronics, HMIs, sensors, actuators, and even flexible energy storage. Complex gel structures and gelation mechanisms regulate the interesting and, in some cases, unique features of MXene hydrogels, necessitating in-depth exploration and engineering at the nanoscale. A simple approach influenced by biomineralization has been developed for the synthesis of multi-functional mineral MXene hydrogels (MMHs) [152]. Wearable tensile strain sensors exhibiting a super-wide sensing range with exceptional sensitivity can be fabricated using the produced MMHs due to their stretchability, self-healing, and conductivity. Strain sensors based on MMHs are intended to be applied to the skin with the overall goal of detecting both small and big movements. To create mechanically durable and sensitive double networked hydrogel strain sensors, MXenes were implemented into a polyacrylamide-sodium alginate soft matrix [153]. The exceptionally high tensile sensitivity in the hydrogel sensor was produced by using highly orientated MXene-based three-dimensional conducting networks. Strain sensors based on MXene hydrogels showed a low hysteresis in their electromechanical performance, as well as the usual frequency-independence characteristic and a fast reaction time.

**Fig. 8:**
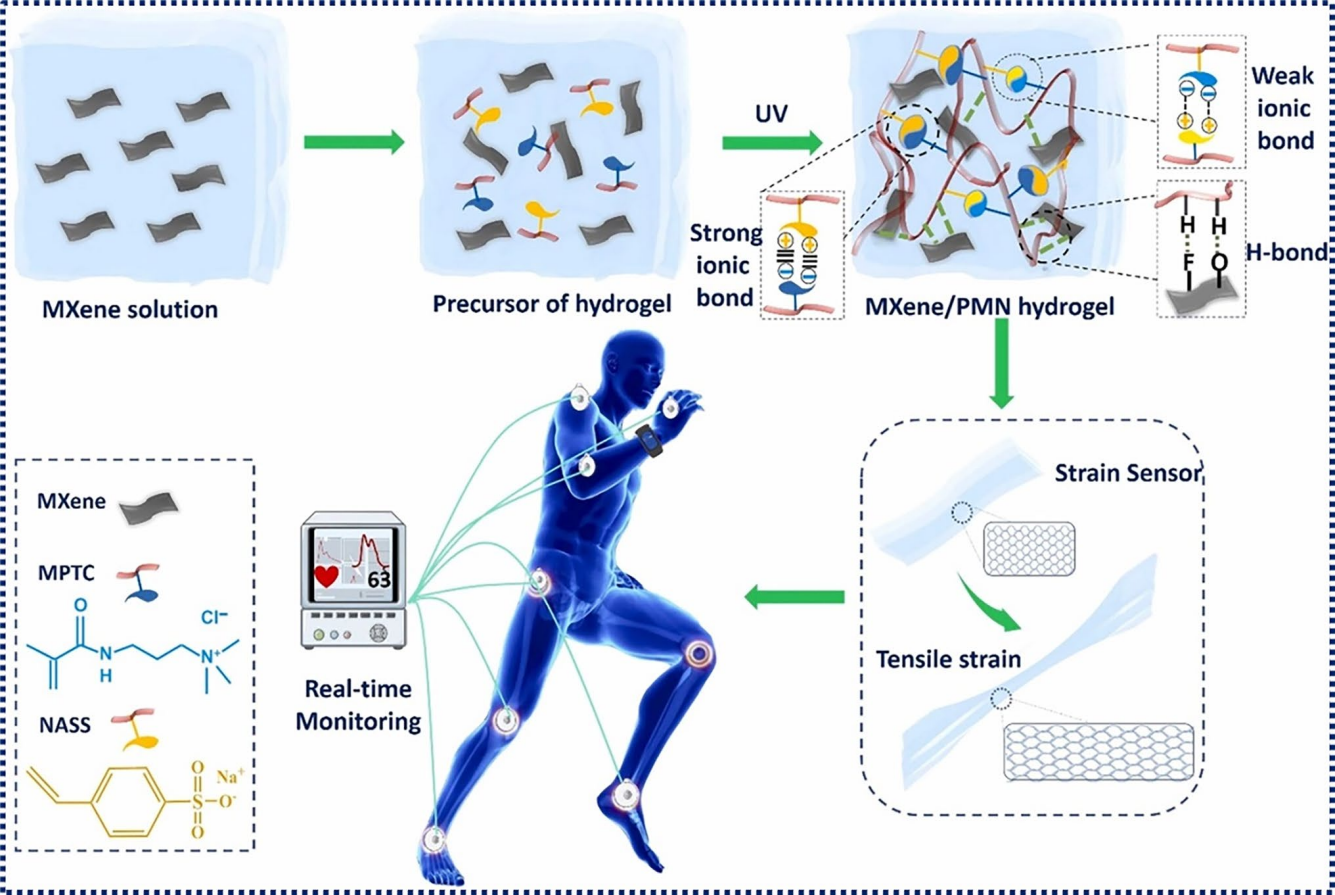
Diagrammatic representation of the manufacturing process for MXene/PMN hydrogels. Reproduced with the permission from Ref. [154], Copyright Elsevier 2022

Hydrogels made of MXenes and polyampholytes (Fig. 8) have also been proposed as a multi-purpose material [154]. This MXene/polyampholytes combination has been shown to be highly effective as a wearable epidermal sensor for the detection of human movements. Assembling this epidermal sensor with the detector of an ADHD patient’s daily activity monitor might help in the treatment of the illness by prompting patients to pay closer attention to their behaviour.

The hetero-structured configuration of the e-skin system proposed by Cai et al. connects vinyl-hybrid-silica nanoparticle (VSNP)-modified polyacrylamide (PAM) hydrogels with MXenes through nano-bridging layers of polypyrrole nanowires (PpyNWs) [155]. The e-skin’s remarkable operating range (2800%), ultrafast responsiveness (90 ms) and robustness (240 ms), strong linearity (800%), customizable sensing mechanisms, and great repeatability result from the multi-dimensional configuration. Cellulose/MXene hydrogels have been initially investigated as strain sensors and EMI shielding because to their biocompatibility, elasticity, and compressibility [156]. Regarding their use as strain sensor, the cellulose/MXene hydrogels demonstrate an excellent conductivity, remarkable stretchability, compressibility, and biocompatibility. This allowed the strain sensor to be sensitive (GF = 6.97), as well as highly stretchable (144.4%), compressible (68.7%), and stable (1000 cycles). Its top-notch functionality enabled to track complex human movements in real time, identify individuals, and track how their bodies are subjected to force. The electromechanical characteristics of the M-hydrogel were studied by synthesising MXene-polyacrylic acid (PAA)/PVA hydrogels to elucidate how the interior ions react to various stimuli, including pH and mechanical strain [157]. They developed a strain sensor based on MXene-PAA/PVA hydrogel, which is safe for human skin, using Bluetooth to remotely monitor electromechanical signals. Since muscle exhaustion is known to cause a shift in sweat pH, a sensing system was developed with this knowledge in mind. Polyacrylamide-co-acrylic acid/chitosan/MXene hydrogels prepared by a simple one-pot free radical polymerization presented a good adaptability to multiple deformation circumstances (tension/bend/compression) and broad temperature adoptability with consistent repeatability. Furthermore, by simply tweaking the glycerol/water ratio, these hydrogels demonstrated remarkable structural stability and dependability throughout a broad temperature range of 20–80 °C [158].

The term “wearable bioelectronics” describes electronics that may be worn on the body to monitor or measure different physiological signals and then communicate that data to another device or platform for further analysis or tracking. Sensors, electrodes, and other components may detect and record biometric data like heart rate, blood pressure, temperature, and more. These devices are frequently designed to be non-invasive and easy to use. The advent of wearable bioelectronics promises a sea change in medical practise by making it possible to monitor one’s health in real time, thus facilitating the early detection and treatment of disease, as well as the provision of more individualised and preventative services. Their usage in scientific studies to collect massive volumes of non-invasive data on human physiology is also on the rise. A technique was developed to efficiently synthesize (20 min) a self-assembled poly-acrylic acid (PAA) hydrogel catalysed by MXenes with high conductivity, high stretchability (1400%), and high resistance to aggregation [159].

The strong, reproducible, and removable adhesion characteristics of TiO_2_@MXene-PAA hydrogels offer substantial benefits when used as non-irritating self-adhesive bioelectrodes. The first dispersion-enhanced MXene hydrogel (DEMH) built by Liu et al. using a chitosan-induced self-assembly approach displayed both exceptional conductivity and sensitivity due to the presence of a highly interconnected 3D MXene@Chitosan network providing rapid transport pathways for electrons [160]. Due to electrostatic self-assembly and hydrogen bonding, MXenes were uniformly dispersed in the polyacrylamide hydrogel, giving the sensor an excellent electrical conductivity (4 × 10^–4^ S cm^−1^) and sensitivity (gauge factor, 11).

The field of wearable sensors has recently exploded around double network (DN) conductive hydrogels. MXene-composed polyvinyl alcohol/sodium carboxymethylcellulose (PVA/CMC) DN hydrogels were shown to be a high-performance strain sensor [161]. Due to their hydrophilic functional groups, MXenes can establish conductive channels and produce a homogeneous distribution in the hydrogel, which is useful for obtaining both a high sensitivity and a large operation window. The distinctive structure and multiple synergistic networks of PCTM give it a favourable mechanical strength (a fracture tensile strength of 1.8 MPa at a fracture strain of 740%), high sensitivity with a wide detection window (a gauge factor of 2.9 at a strain range of 0–700%), and long-term durability over 3000 continuous cycles.

A physically robust and conductive MXene gelatin organo-hydrogel with good environmental stability and self-adhesiveness was developed by Wang et al. for the construction of biodegradable multifunctional sensors [162]. The organo-hydrogel showed a high conductivity due to MXenes’ incorporation, which correlated with the relative MXene content. Ti_3_C_3_T_*x*_ helped to initiate the rapid gelation of a variety of polymeric hydrogels within a few minutes, beginning with monomers [163]. Mechanical strength, adhesion, as well as self-healing were improved when MXenes was used as cross-linkers. A polymer-MXene hydrogel was shown to exhibit unique thermosensation-based actuation upon near-infrared illumination, accompanied by rapid shape transformation due to the combination of the photothermal behaviour of Ti_3_C_3_T_*x*_ and the heterogeneous phase-transforming of the involved polymers. Hydrogels containing MXenes, a physically cross-linked PAM network, and gelatin were used to create multi-functional, wirelessly transmittable sensors with high sensitivity, great mechanical strength, and great pliability [164].

**Fig. 9:**
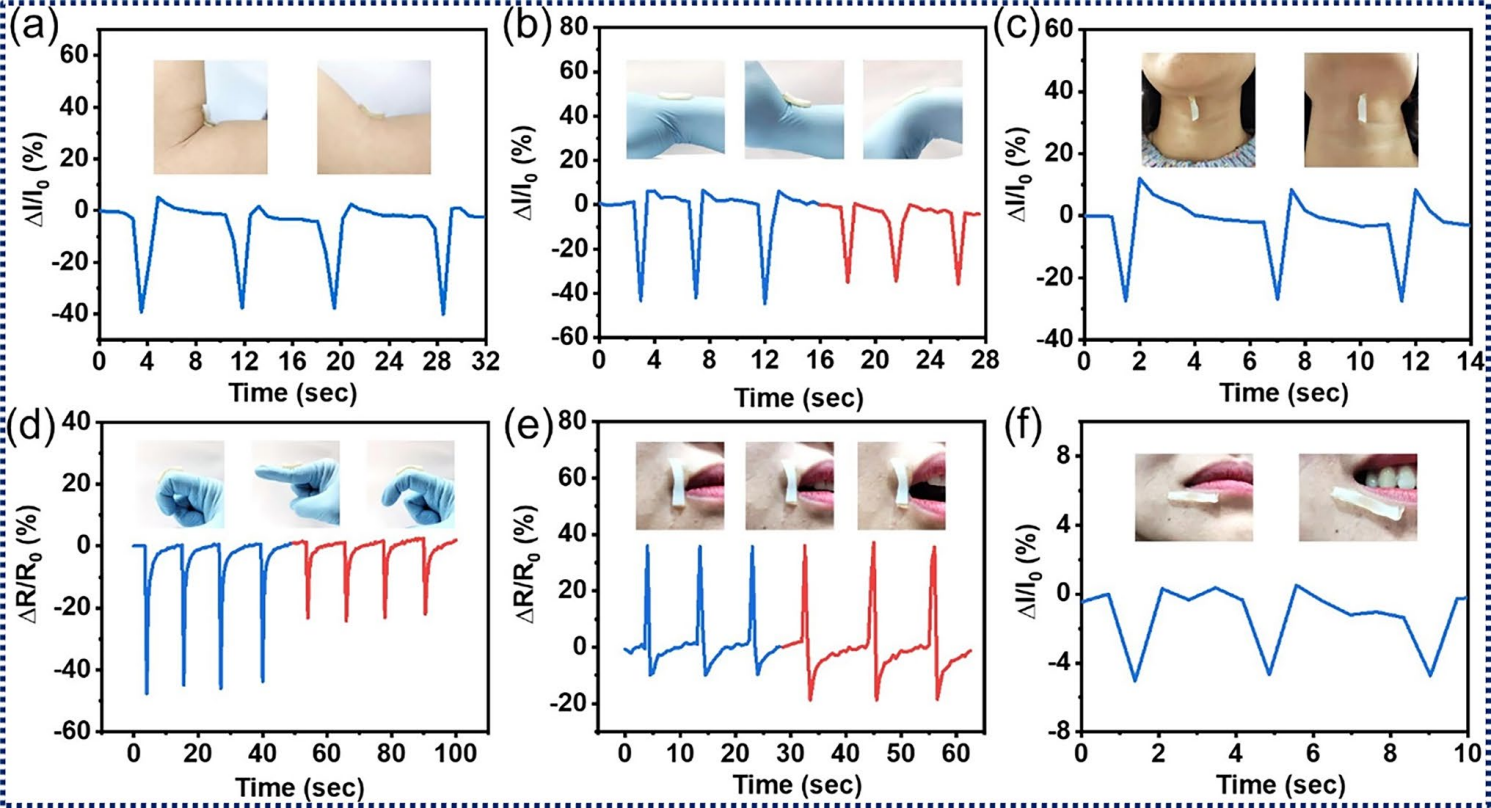
Human motions detection. **a** How the bilayer composite hydrogel strain sensor’s electrical signal changes when the arm bends. **b** When the wrist moves in two different ways, the relative current of the strain gauge changes. **c** How the piezoresistive reaction of the strain sensor changes when the head moves. **d** The electrical output from the strain sensor when the finger is bent 45° and 60°. **e** When the mouth opens, there is a change in the relative resistance of the strain gauge. **f** Response curves for smiling right now. Reproduced with the permission from Ref. [3] Copyright Elsevier 2021

Wearable flexible sensors that can be worn have shown great promise in underwater robots and ocean exploration. These monitors can be placed on the outside of underwater vehicles or stuck to the bodies of aquatic animals to keep track of how they move and how they feel. Flexible sensors are good to be used underwater because they can bend into odd forms and stand up to the harsh conditions of the sea. These gauges can sense temperature, pressure, and the chemical make-up of a substance. The underwater systems can be combined with wireless transmission and machine learning algorithms with the overall goal to check on the health of coral reefs, track the moves of marine animals, or find oil spills underwater. MXene and polyhydroxyethyl methacrylate (PHEMA) have been used to fabricate and formulate hydrogels [165]. In this regard, MXene/PHEMA hydrogels have a big sensing range (more than 400%), a high tensile strength (0.54 MPa), a skin-compliance modulus (120 kPa), high sensitivity (4.42 at 150–450% strain), short response time (200 ms), and can sense for a long time (over 1000 cycles) [165]. With their ability to not grow and not stick to other things, the hydrogels were able to keep their high mechanical strength and cyclic stability after being in water for 30 days. They could also accurately track the movements of human and shark models underwater. It was possible that MXene/PHEMA hydrogels that don’t swell and don’t attract dirt could be used to make electrical skins for harsh liquid environments. There is a high demand for flexible electronics with superior electromechanical and biotherapeutic qualities to properly monitor human physiological processes and to aid in wound healing. Using a sequential in-situ polymerization method, a bilayer composite hydrogel with excellent stretchability, durability, sensitivity, and strong adherence, which made it ideal for the use on human skin was developed [3]. The wide variety of activities that can be detected by the hydrogel strain sensor from body movements is seen in Fig. 9. Wide-ranging potential exists in limb control, due to the strain sensor’s steady and dependable electrical signal output even under massive deformations of the human body.

### Biopolymer/MXenes-Based Stretchable Sensors

The unique properties of biopolymers such as reversible flexibility, renewability, biocompatibility, biodegradability, and self-healing ability have garnered significant interest in the field of wearable and stretchable sensors [166]. The development of biopolymer/MXene-based composites has facilitated the design and fabrication of sensitive and wide-range stretchable sensors. Natural biopolymers mainly include cellulose, silk fibroin (SF) and chitin/chitosan (CS) [166], among which cellulose is the most commonly utilized to prepare biopolymer/MXene-based composites for stretchable sensors.

**Fig. 10:**
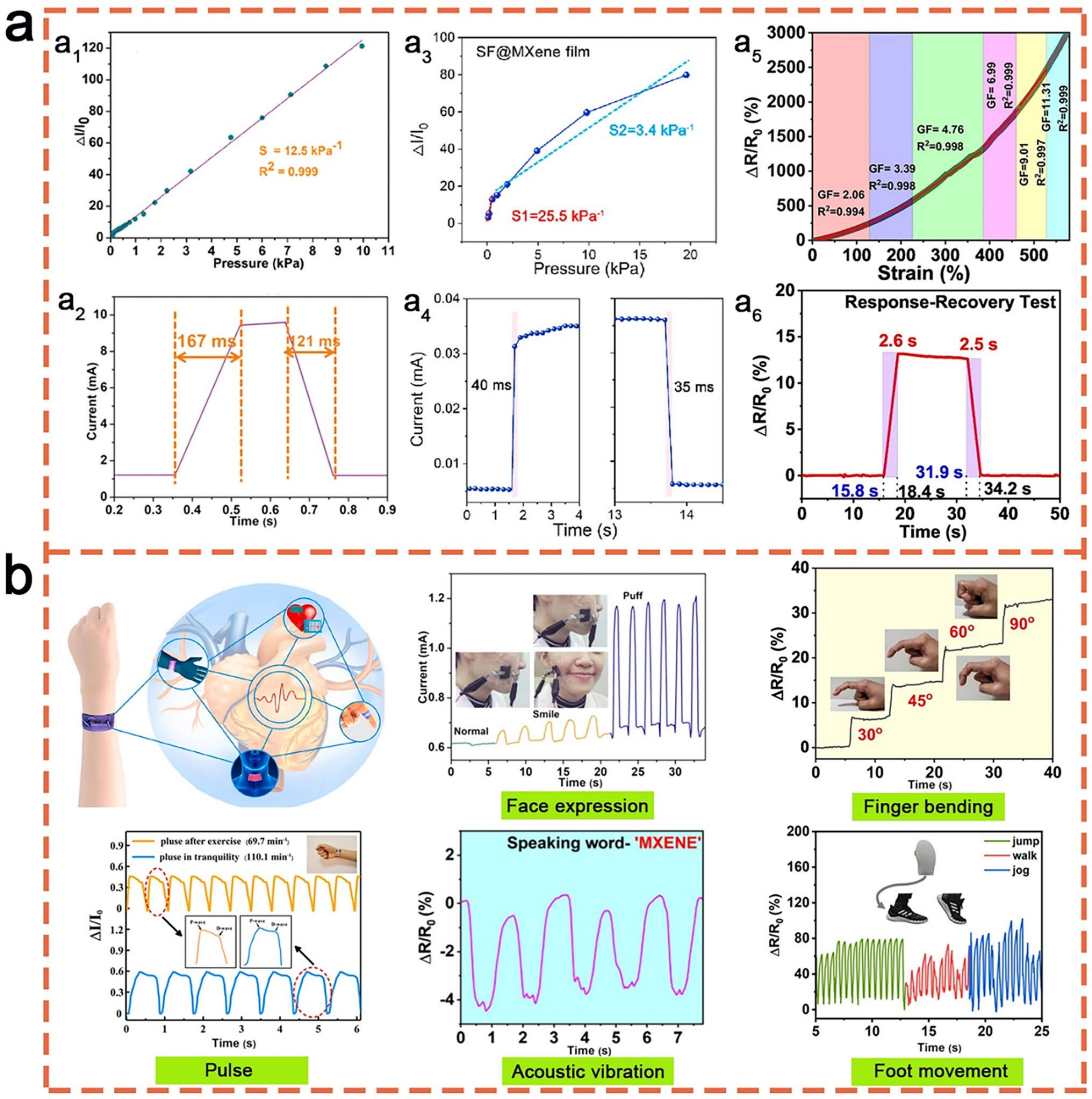
**a** Sensitivities (**a**_**1**_, **a**_**3**_, **a**_**5**_) and response times (**a**_**2**_, **a**_**4**_, **a**_**6**_) of MXene/CNF. Reproduced with the permission from Ref [167]. (Copyright American Chemical Society, 2019), MXene/SF [172] (Copyright Elsevier, 2020) and MXene/CS [177] (Copyright Elsevier, 2023) based pressure sensors, as well as **b** their applications in human health detections, such as face expression [167] (Copyright American Chemical Society, 2019), finger bending [177] (Copyright Elsevier, 2023), pulse measurement [168] (Copyright Elsevier, 2021), acoustic vibration [177] (Copyright Elsevier, 2023), and foot movement [176] (Copyright Elsevier, 2023)

Chen et al. [167] successfully fabricated a light-weight, compressible and elastic carbon aerogel (CECA) through connecting Ti_3_C_2_T_*x*_ MXenes using bacterial cellulose (BC). The developed CECA demonstrated a board range of possible working pressures between 0 and 10 kPa, a high linear sensitivity of 12.5 kPa^−1^ (0–95% strain, Fig. 10a1), fast response-recovery times (Fig. 10a2), and an ultra-low detection limits of 1.0 Pa. Furthermore, it exhibited an ultra-high structural stability, which withstood a long-term compression at 50% strain for at least 100,000 cycles (Fig. 10a, b). Su et al. [168] reported an ultra-high sensitive piezoresistive sensor based on Ti_3_C_2_T_*x*_/cellulose nanofiber (CNF) foams. This sensor exhibited sensitivities as high as 419.7 and 649.3 kPa^−1^ in the stress ranges of 0–8.04 and 8.04–20.55 kPa, respectively, along with a low detection limit of 4.0 Pa. Yang et al. [169] developed a pressure sensor based on Ti_3_C_2_T_*x*_/BC nanofiber, which showed an improved detective limit of 0.4 Pa and a fast response time of 95 ms.

Recently, more studies have been devoted to develop biopolymer/MXene-based hybrid architectures to further improve the performance of the scratchable sensor. To expand the sensing range of biopolymer/MXene stretchable sensors, Huang et al. [170] designed a hierarchical multi-layer Ti_3_C_2_T_*x*_/CNF sensing layer with a wide detection range of 0–950 kPa. Xu et al. [171] successfully constructed a Ti_3_C_2_T_*x*_/carbon nanotube (CNT)/CNF architecture. When used as a pressure sensor, an ultra-high linear sensitivity of up to 817 kPa^−1^ was achieved due to its excellent electrical conductivity of ~ 2400 S m^−1^.

SF is extracted from silkworm cocoons and stand out among other biopolymers due to its exceptional mechanical strength and ease of surface modification. MXene/SF bio-composites are usually assembled through 3D cross-linking of MXenes, SF, and/or other compounds to synthesize sensing materials. Wang et al. [172] developed a flexible Ti_3_C_2_T_*x*_/SF bio-composite film with a 3D cross-linked structure, employing natural SF as a bridging agent. This film demonstrated a comparable sensitivity to MXene/CNF composites under low pressure but exhibited relatively short response/recovery times of 40/35 ms (Fig. 10a3) but exhibited relatively short response/recovery times of 40/35 ms as depicted in Fig. 10a4. Inspired by the multi-scale architecture of nacre, Abadi et al. [173] fabricated a series of Ti_3_C_2_T_*x*_/SF/graphene oxide (GO) composite foams with varying MXene/SF ratios through compositing glutaraldehyde (GA)-cross-linked SF, GO and MXenes. The sensitivity of the MXene/SF/GO composite surpassed that of MXene/GO composites [174] and could be adjusted by modifying the MXene/SF ratio. Paolieri et al. [175] successfully prepared a library of materials based on Ti_3_C_2_T_*x*_/SF/tannic acid (TA) composites. The MXene/SF/TA gums exhibited an impressive stretchability of 600 times couple with a promising self-healing ability. When varying the strain from 20 to 100%, the sensitivity of the MXene/SF/TA pressure sensor remained almost unchanged, while the highest gauge factor was about 0.59. To improve the sensitivity of the MXene/SF based sensor, Yang et al. [176] fabricated an oriented Ti_3_C_2_T_*x*_/SF/hyaluronic acid (HA) hydrogel. The resulting gauge factor steadily increased from 4.5 at 5% strain to 11.3 at 80% strain, while non-oriented MXene/SF/HA hydrogel only reached a maximum gauge factor of 5.7 (65% strain).

CS, the deacetylated derivative of chitin, does not only possess favourable biological properties but also presents an improved solubility, an excellent processability and highly sophisticated functionality. These characters make MXene/CS composites highly applicable in the field of stretchable sensors [158, 177] (Fig. 10a, b). Ti_3_C_2_T_*x*_/CS/poly(acrylamide-acrylic acid) (PA) hydrogels developed by Li et al. [158] combined an excellent mechanical performance with an enhanced electrical conductivity, a stable temperature tolerance, and a rapid self-healing ability. These advantages position MXene/CS/PA hydrogels as a promising sensing material. Importantly, the MXene/CS/PA strain sensor has an outstanding sensitivity in a wide temperature range of -20–80 °C. In virtue of their outstanding and adjustable sensing performance, which is summarized in Table 4, biopolymer/MXene-based stretchable sensors have been widely applied in the applications of human health detections, such as face expression, finger bending, pulse, acoustic vibration, and foot movement (Fig. 10b).

**Table 4:** Performance of the biopolymer/MXene-based stretchable sensors

Sensing materials	Sensor type	Sensitivity	Sensing range; detection limit	Stability; response time	References
MXene/BC	Pressure sensor	12.5 kPa^−1^ (0–10 kPa)	0–10 kPa; 1.0 Pa	100 cycles (99% strain), 100,000 cycles (50% strain); 167 ms	[167]
MXene/CNF		419.7 kPa^−1^ (0–8.04 kPa), 649.3 kPa^−1^ (8.04–20.55 kPa)	0–20.55 kPa; 4.0 Pa	10,000 cycles; 123 ms	[168]
MXene/CNF		95.2 kPa^−1^ (2–50 Pa), 27.5 kPa^−1^ (0.05–3 kPa), 0.94 kPa^−1^ (3–10 kPa)	2–10 kPa; 0.4 Pa	25,000 cycles; 95 ms	[169]
MXene/CNF		10.7 kPa^−1^ (0–240 kPa), 34.6 kPa^−1^ (240–640 kPa), 16.6 kPa^−1^ (640–950 kPa)	0–950 kPa; -	3000 cycles; 298 ms	[170]
MXene/CNT/CNF		817 (0–0.2 kPa), 234.9 (0.2–1.5 kPa)	0–1.5 kPa; -	2000 cycles (30% strain);74 ms	[171]
MXene/SF		25.5 kPa^−1^ (0.1–0.5 kPa), 3.4 kPa^−1^ (1.0–20 kPa)	0–20 kPa; 9.8 Pa	3500 cycles (73 Pa); 40 ms	[172]
MXene/SF/GO		14.23 kPa^−1^ (MXene/SF (9:1), 1.4 kPa), 12.53 kPa^−1^ (MXene/SF (5:1), 1.4 kPa),	0–1.5 kPa; 72 Pa	-	[173]
MXene/SF/TA		GF: 0.59	20–100% strain; -	600 cycles (self-healing); -	[175]
MXene/SF/HA	Strain sensor	GF: 4.5–11.3 (5–80% strain)	–	10,000 cycles (50% strain); -	[176]
Mxene/CS/PA	Strain sensor	GF: 3.15 (− 20 °C), 3.93 (25 °C), 2.55 (80 °C)	1–600% strain, 0.08–3.2 kPa stress; 0.5 g	-; 500 ms	[158]

### Rubber/MXene-Based Stretchable Sensors

**Fig. 11:**
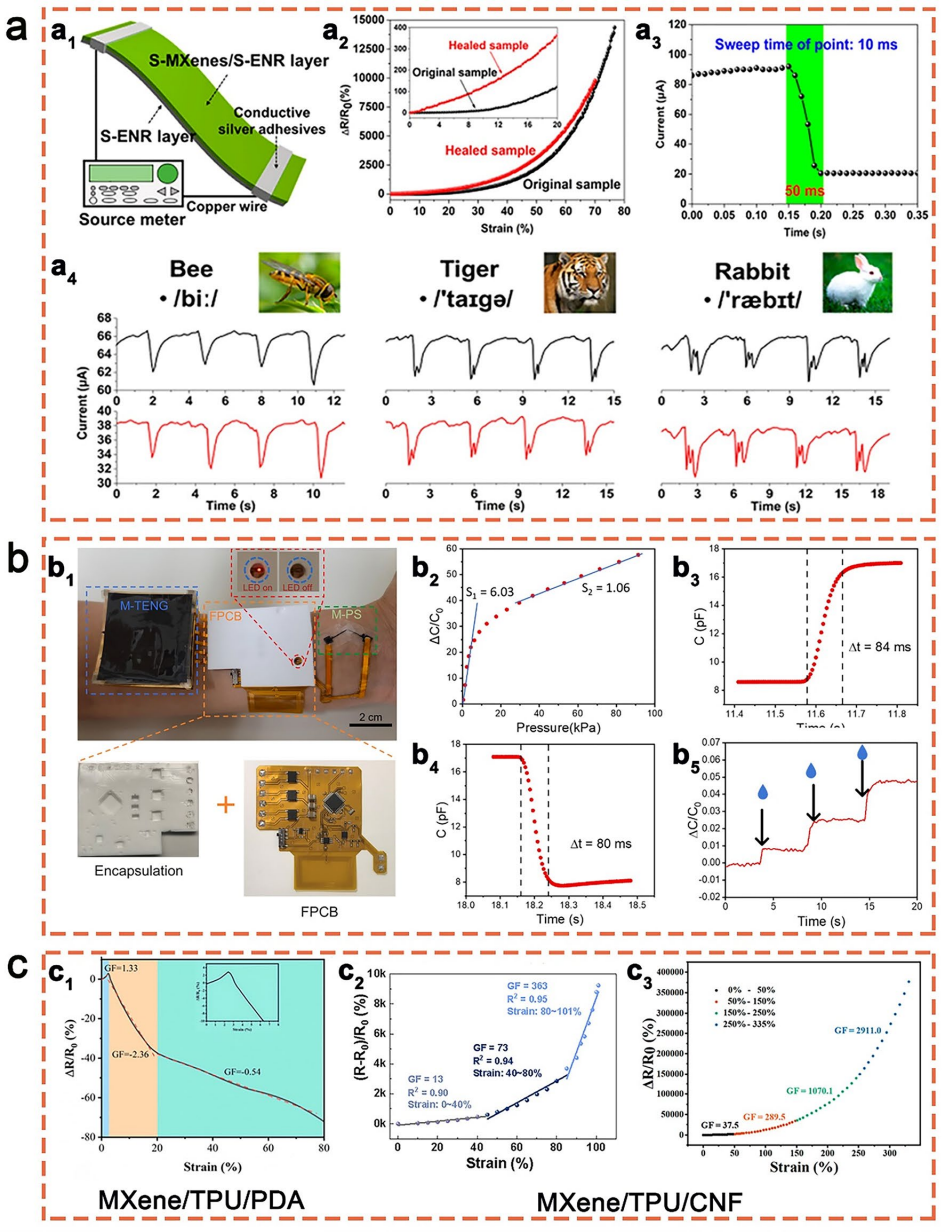
**a** Structure (**a**_**1**_), properties (**a**_**2**_, **a**_**3**_), and speech detection application (**a**_**4**_) of the Ti_3_C_2_T_*x*_/ENR stretchable sensor. Reproduced with permission from Ref [178]. (Copyright American Chemical Society, 2020). **b** Optical image (**b1**), and sensing performance (**b2**–**b5**) of the self-powered Ti_3_C_2_T_*x*_/SEBS sensing system. Reproduced with permission from Ref [184]. (Copyright Elsevier, 2022). **c** Comparison of gauge factor between MXene/TPU/PDA and MXene/TPU/CNF stretchable sensors. Reproduced with permission from Refs [181–183]. (Copyright Elsevier, 2022), (Copyright American Chemical Society, 2022), (Copyright American Chemical Society, 2021)

For the sensors subjected to repeated stress, rebound stability is a critical property to achieve high performance and to extend their service life. Rubbers, known for their elasticity, toughness and ability of reversible deformation, have emerged as promising materials for MXene-based sensors. For instance, Guo et al. [178] developed a Ti_3_C_2_T_*x*_/epoxidized natural rubber (ENR) stretchable sensor, for which MXenes were modified by serine as shown in Fig. 11a. The MXene/ENR sensor exhibited a high gauge factor (107.3), a low strain detection limit (0.1%) and a fast response time (50 ms) for human motion, as well as an excellent self-healing ability.

Besides natural rubber, synthetic rubbers such as styrene-butadiene rubber (SBR) [179], polydimethylsiloxane (PDMS) [145, 180] and thermoplastic polyurethane (TPU) [181–183], have been widely applied in rubber/MXene stretchable sensors. Li et al. [179] fabricated Ti_3_C_2_T_*x*_/SBR nanocomposites through freeze–drying and mechanical mixing. In addition to an improved thermal conductivity, the sensitivity of the developed MXene/SBR nanocomposites was well recovered during stretching and shrinking cycles under large strains ranging between 70 and 100%. Yi et al. [184] prepared a self-powered Ti_3_C_2_T_*x*_/Styrene-ethylene-butylene-styrene (SEBS) sensing system comprising power-efficient triboelectric nanogenerators (TENG), highly sensitive pressure sensors, and multi-functional circuitry (Fig. 11b). Zhang et al. [145] reported a Ti_3_C_2_T_*x*_/PDMS stretchable strain sensor with a gauge factor of 3.6 for the entire stretching process. Moreover, the reversibility of multiple hydrogen and imine bonds in the Ti_3_C_2_T_*x*_/PDMS composite improved its tensile properties and self-healing ability without external stimulation.

To expand the working range and improve the sensitivity of sensing materials, MXene and rubber have been hybridized with other functional sensing materials, such as polydopamine (PDA) and carbon nanotubes (CNTs). Ti_3_C_2_T_*x*_/TPU/PDA sensors [181] demonstrated an excellent flexibility, a fast response time of ~ 40 ms, a good stability over 5000 cycles, a wide range of 10 Pa–122.5 kPa and a gauge factor up to 2.36 (2.5–10% strain). Regarding improvements of the resulting gauge factor, MXene/rubber/CNT sensors showed a significant advantage. Ti_3_C_2_T_*x*_/TPU/CNT sensors developed by Wang et al. [183] and Dong et al. [182] possessed high sensitivities with gauge factors of 363 and 2911, respectively. Figure 11c illustrates the comparison of the gauge factor between Ti_3_C_2_T_*x*_/TPU/PDA and Ti_3_C_2_T_*x*_/TPU/CNT sensors.

### MXene-Coated Functional Textile-Based Sensors

Textiles are a good place for sensors to be mounted because they are light, bendable, and can be shaped to fit into wearable devices. Moreover, textiles are easy to wear and allow air to pass through, which is important for medical monitoring and sport performance. Functional fabrics are made of materials that can be used to make electronics and devices that can be worn. By putting them together, MXenes-coated functional textile-based devices can be created.

To create effective textile-based devices coated with MXenes, MXenes are deposited on the textiles by spin coating, drop casting, or screen printing. After MXenes deposition, the surface of the cloth is patterned with wires to make a sensor. A temperature sensor made of MXenes-coated polyester cloth and silver electrodes is an example of a useful textile-based MXene sensor [185]. It was shown that the sensor was sensitive to changes in temperature with reaction times below 1 s. A gold electrode and MXene-coated nylon cloth were used to make a strain gauge, which monitored strains as small as 0.5%. The high sensitivity and specificity of textile-based MXene-coated sensors is another benefit. It has been shown that MXenes are good at conducting electricity, which makes them useful in monitors that look for changes in electrical qualities.

Moreover, MXenes can be modified with different chemical groups, which makes it possible to selectively detect certain analytes. Liu et al. showed how to make electrically conductive and superhydrophobic silk materials for EMI shielding and humidity tracking by vacuum-assisted layer-by-layer assembly [186]. The leaf-mimicking nanostructure was made of silver nanowires (AgNWs) acting as highly conductive skeleton (vein) and transition metal carbide/carbonitrides as the leaf’s skin (lamina). The presence of MXenes helped to prevent the oxidation of AgNWs and made it easier for them to bond with the cloth substrate.

Changing the MXenes’ functional groups increased the water repellence of the sensor. Cao et al. used 3D printing to create multi-purpose fibres and fabrics with excellent photo-thermal, electro-thermal, and electromechanical response [187]. MXenes have been investigated for their potential to strengthen synthetic fibres due to their fascinating physical/chemical features. Strain sensors with high sensitivity can be made from textiles with electromechanical capabilities such as Ti_3_C_2_ coated cellulose nanofibers.

**Fig. 12:**
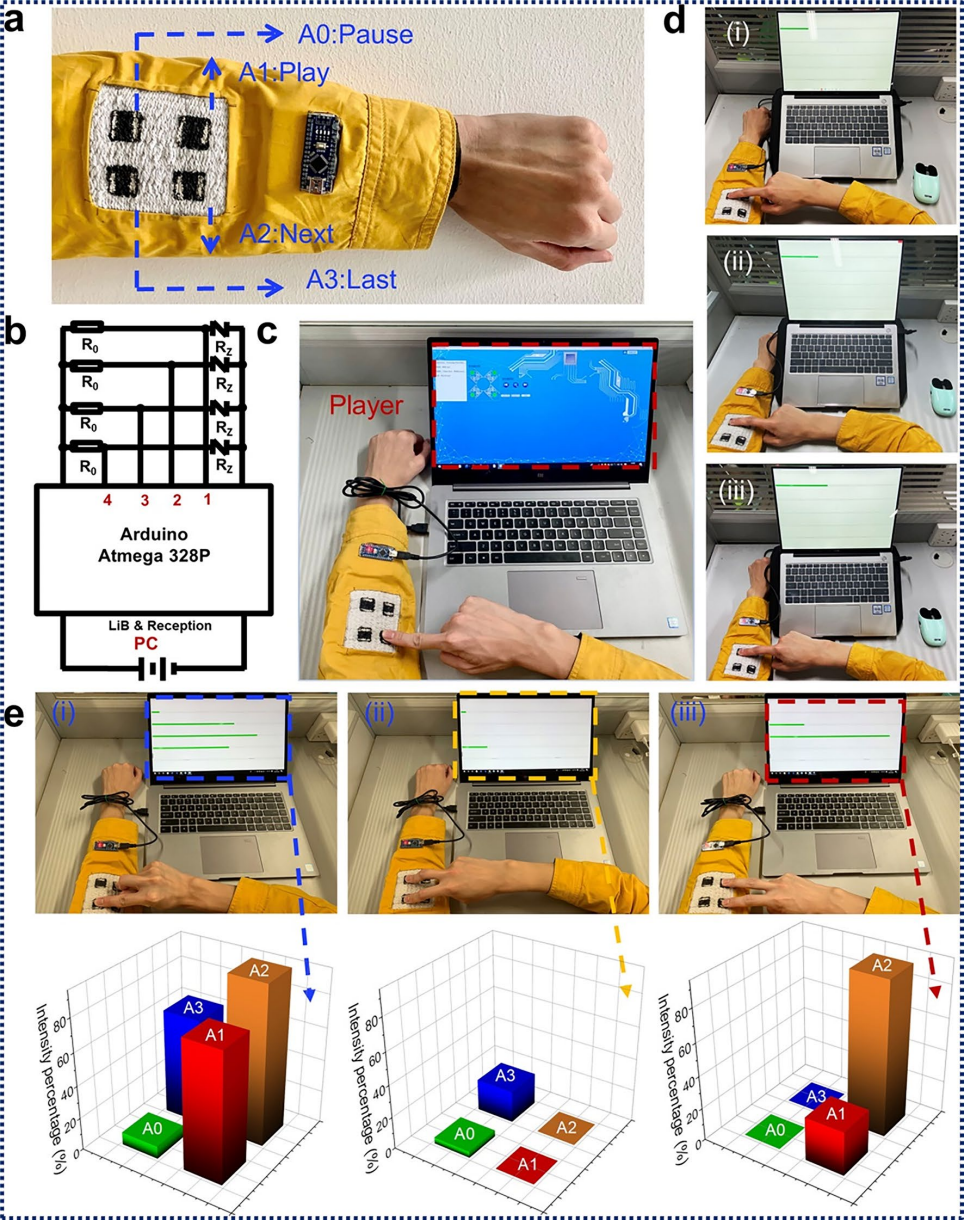
Proof-of-concept for HMI systems made with MXene-coated cotton. **a**, **b** Digital picture and circuit layout of the MXene-coated cotton pressure-sensing fabric interface. **c** Software for using MXene-coated cotton pressure-sensing cloth, called MXene Tools. **d**, **e** MXene Tools pressure sensing (single area and multi-region) and pressure-sensitive data modelling diagrams in 3D. Reproduced with the permission from Ref. [4], Copyright American Chemical Society 2020

Li et al. presented a piezoresistive pressure sensor using dip-coated MXene-textiles. The sensor’s high sensitivity (12.095 kPa^−1^ for the range 29–40 kPa and 3.844 kPa^−1^ for the range < 29 kPa) was traced back to the combination of MXenes’ good electrical characteristics and the cotton’s wavy surface [188]. The MXene-textile pressure sensor identified the wearer’s speech as well as monitor wrist pulse waves and finger motions. Luo et al. built a multi-core shell structure to create a smart textile that is both waterproof and breathable [189]. The elastic polydopamine (PDA) coated cloth was decorated with MXenes before being coated with PDMS. The MXene coated fibres functioned as the conducting network, and the PDMS coating could do both tasks by insulating the MXene from oxidation while also providing superhydrophobicity and, by extension, corrosion resistance to the textile. The photo-thermal and electro-thermal conversion performance of the smart textile was exceptional and long-lasting. By studying adsorption and binding capabilities, the interactions between MXene and textiles were revealed. Cotton’s naturally rough surface aided in the solid adsorption of MXenes. As a result, despite mechanical washing and ultrasonic treatment, MXene was difficult to separate [4]. As a flexible pressure sensor, this MXene-coated cotton fabric demonstrated a high gauge factor (7.67 kPa^−1^), quick reaction and relaxation speed (35 ms), outstanding stability (> 2000 cycles), and remarkable washing durability due to its force-sensitive resistance. The team linked the MXene-coated cotton pressure sensor in series to an Arduino analogue transmission circuit, as shown in Fig. 12, which translated the pressure-induced current changes into digital signals to investigate the fabric’s potential practical applications.

#### MXene-Based Pressure Sensing Mechanisms

Pressure sensors can be classified into piezoresistive, capacitive, piezoelectric, triboelectric, and potentiometric types depending on their overall working principle. However, the necessary material properties for the sensors change with their operating principle. Because of their superior properties, MXenes have been used in multiple sensing techniques. When MXenes are employed in a pressure sensor with a particular working principle, the respective performance must be optimized.

##### Piezoresistive

In this case, the pressure sensors primarily depend on the resistance variation of the pressure sensing layer. MXene-based piezoresistive sensors have received notable attention due to their high performance and simple structure, operating mechanism, and fabrication process. These sensors benefit from the pressure-adjustable MXene layer spacing and tunable conductivity. Therefore, when MXenes are used in piezoresistive sensors, they are primarily utilized as a force-sensing layer, with an emphasis on optimizing the structure of the force-sensing layer to boost and enhance the force-sensing performance. The sensing mechanism of Ti_3_C_2_T_x_/rGO aerogels, as proposed by Ma et al., was attributed to the contact between its components, which is influenced by compression [174]. Under the application of an external force, the sensor experienced a reversible compressive deformation. This structural modification resulted in more conductive filler contact in the aerogels, leading to more conductive pathways. Consequently, the resistance between the components reduced, thus decreasing the overall resistance of the aerogel. Therefore, the change in conductivity for the piezoresistive layer with a porous structure is attributable to the increase/decrease of the conductive routes generated by the application/release of the external pressure. Su and Yue et al. have also proposed a similar compression recovery mechanism for hybrid Ti_3_C_2_T_x_/CNF-foams and Ti_3_C_2_T_x_-sponges [168, 190]. Among various sensors, the piezoresistive sensor based on Ti_3_C_2_T_x_/CNF-foams exhibited an exceptionally high sensitivity of 649.3 kPa^−1^ within a pressure range of 8.04–20.55 kPa. Additionally, this sensor demonstrated a rapid response time of 123 ms and a remarkable durability, withstanding up to 10,000 cycles. The piezoresistive sensor based on Ti_3_C_2_T_x_ sponges exhibited a sensitivity of 442 kPa^−1^ within the pressure range of 5.37–18.56 kPa and rapid response time of 138 ms.

##### Capacitive

The capacitive pressure sensor acquires advantages from the change in capacitance induced by an external mechanical pressure. The optimization of the performance of these sensor types often involves the adjustment of the distance between the electrodes on both sides, area, and the dielectric constant of the material. MXenes are used as an electrode of capacitive sensors or to construct microstructures to produce a variable area under pressure due to their flexibility and high conductivity. For instance, Uzun et al. developed a capacitive textile sensor device with Ti_3_C_2_T_x_-coated conductive yarn braided fabric as both-end electrodes and a thin nitrile rubber dielectric layer sandwiched between them [191]. The resulting sensor capacitance increased with compressive strain and returned to its initial value when the external force is removed. This capacitive textile sensor exhibited a high level of sensitivity, with a gauge factor of around 6.02. It also possessed a broad sensing range (compression of up to 20%) and demonstrated an exceptional cycling stability of 2000 cycles with a compression strain of 14%.

##### Piezoelectric

The piezoelectric pressure sensor is based on the properties of piezoelectric materials. The external force causes the internal charges of the material to be unevenly distributed, causing in charge polarization, which can be removed by eliminating the external force. Typically, the optimization of piezoelectric sensors focuses on the piezoelectric properties of reinforced materials. Previous research has indicated that MXenes possess a non-central symmetric lattice structure and exhibited anisotropic piezoelectric properties [192]. Furthermore, to enhance their piezoelectric characteristics, MXenes can be combined with piezoelectric polymers, including polyvinylidene fluoride (PVDF) and polyvinylidene fluoride trifluoroethylene (PVDF-TrFE) [193, 194].

##### Triboelectric

Triboelectrification is fundamentally characterized by the charge transfer between two contacting materials with different electron attraction capabilities. Due to their exceptional flexibility and elevated conductivity as well as excellent durability, MXenes are frequently utilized in triboelectric sensors as a flexible electrode or electron donor [195]. Salauddin et al. reported the use of a double-side-contact (DSC) triboelectric nanogenerator (TENG) operating under the contact-separation mechanism [196]. The TENG’s top and bottom layers were composed of human skin, serving as the positive material, as well as a combination of MXene and silicon, acting as the negative material. Additionally, MXene-coated fabric was employed as charge trapping layers in the TENG system. The primary operational principle can be described as follows: In the absence of any external force acting upon the DSC-TENG, no charge transfer will occur. Upon applying additional pressure to the device surface manually, the material surfaces of each layer came into contact with each other. Furthermore, an equal quantity of positive and negative charges accumulated on the surfaces of the nanocomposites, which possess varying electron attraction capabilities. Upon gradually releasing the pressure, the human skin and the surface of the MXene/silicone nanocomposites underwent a gradual movement and separation as well as a subsequent passage of positive charge from the human skin to the electrode. This process demonstrated that, during pressure release, the electrostatic induction effect generated a significant potential difference between the friction layers. Consequently, a substantial positive charge accumulated on the surface of the microstructure attached to the common single electrode. Consequently, free electrons were capable to flow through the external circuit from the electrode. When the external pressure was completely released, the friction layer returned to its original state. Based on the composite material of MXene and silicon, the triboelectric sensor demonstrated a maximum power density of 55.47 W m^−2^ when applying a load resistance of 0.18 MΩ.

##### Potentiometric

In addition to these traditional pressure-sensing mechanisms, researchers have recently introduced an all-in-one self-powered pressure-sensing mechanism, which is called potentiometric transduction mechanism and based on emerging trends of future miniaturized electronic skin. This method has the benefit of supplying self-energy and producing stable high-frequency/low-frequency electrical signals. It compensates for some of the drawbacks of the previously mentioned pressure systems, since piezoresistive and capacitive pressure sensors require additional lines and energy supply apparatus, whereas piezoelectric and tribo-electric equipment need to utilize specific materials and cannot produce stable static signals.

In this context, the galvanic cell system inspired the potentiometric transduction mechanism. The pressure signals are translated into a sustainable electrical signal output through the utilization of a redox reaction between both sensor electrodes. Because of their flexibility and good functionalities as well as tunability, MXenes can adsorb a wide range of ions. Regarding potential mechanisms, they can be utilized as an electrode for redox reactions. Lei et al. reported an all-solid-state pressure sensing rGM (rGO/GO/PVA nanofibers/MXene) system based on a mechanical potentiometric transduction (MPT) mechanism that transforms mechanical stimulation into a change in the ion transport channel. This translated into a measurable potential difference between two electrodes with hydrated GO, creating a potential conduction behaviour in addition to a programmable stable voltage and current [197]. When subjected to a constant mechanical pressure, the self-powered rGM sensor provided an output open-circuit voltage of 0.58 V and a short-circuit current density of 3.2 A cm^−2^. This work described the innovative concept based on the MPT mechanism that will simplify production while increasing the efficiency of future electronic products and smart systems.

### MPCs for Breath-Based Biomarker Diagnosis

Although the most known biofluids, such as blood, urine, saliva, sweat, and interstitial fluid, are vastly used in biosensing intentions to provide an efficient medicinal diagnostic, myriads of invaluable pieces of information can be derived from the target molecules, which predominantly stay within the exhaled breath. Not only are amounts of various gases comprising the exhaled breath directly associated with the metabolisms inside the human body, which is suitable for early diagnosis, but even breath-based biosensors offer one of the most non-invasive and robust techniques [198].

These gas-phase molecules can be primarily categorized into two main groups, namely volatile organic compounds (VOCs) such as alcohols, aldehydes, and ketones, and non-volatile compounds (non-VOCs) like hydrogen, ammonia, CO_2_, NO_x_, and hydrogen peroxide. Any aberrant concentrations of both VOCs and non-VOCs in exhaled breath imply dysregulated metabolism in different internal organs, leading to discernible pathological conditions [199]. Consequently, it is crucial to promptly detect these biomarkers once their level exceeds the normal range, following a proper administration of medical treatment to preclude the ominous disease. This spurs new innovations towards faithfully monitoring traces of VOCs and non-VOCs in breath of which concentrations are in the range of part per billion (ppb) to million (ppm) [5].

Toxic gas sensors are one of the most significant and essential applications for 2D materials. Yang et al. investigated X_2_CO_2_ MXenes (X = Sc, Ti, Zr, and Hf) as a gas sensor material for NO and CO detection using DFT approaches. Based on their numerical results, it became evident that the NO molecules can be attached to the X atoms. When looking at the X_2_CO_2_-derived MXenes, the bond length between NO and Sc_2_CO_2_ was the shortest, measuring 2.17. The bond lengths between the adsorbed gas and the X atom became shorter for different early transition metals, such as 2.7 for Zr_2_CO_2_, 2.66 for Hf_2_CO_2_, and 2.34 for Ti_2_CO_2_. The adsorption energy of NO is highest for Sc_2_CO_2_ (− 0.47 eV) compared to other MXene-based materials like Ti_2_CO_2_ (− 0.34 eV), Hf_2_CO_2_ (− 0.28 eV), and Zr_*2*_CO_*2*_ (− 0.23 eV). This was traced back to the strong link between the bond length and the adsorption energy [200]. Experimental studies using alkalized V_2_CT_x_ for highly selective sensing behaviour towards NO have been conducted on the basis of theoretical predictions [201]. The electron transport was shown to be enhanced by the presence of free electrons close to the vacant Fermi energy level.

Hydrogen sulphide (H_2_S) is another dangerous gas emitted by most oil and refinery, tanning, and pulp industries. It has a strong, unpleasant odour even at low concentrations, as well as devastating effects on human being [202]. Xu et al. took advantage of MXenes’ gas sensing properties to create a metal oxide-MXene nanocomposite for H_2_S sensing [203]. The exposure to H_2_S resulted in a dramatic rise in resistance, suggesting that molecules of the gas were adsorbed between in interlaminar spacing of the Ag nanoparticles painted on MXene nanosheets. This blocked the movement of charge carriers and prevented electrons from jumping from one atom to another [204].

The non-invasiveness of breath-based biosensors immensely paves the way for individuals toward implementing a low-cost, readily accessible point-of-care (POC) devices compared to other state-of-the-art biosensors that remain mainly inside laboratory and clinical settings [205]. Notwithstanding semiconducting metal oxides [206], graphene and its derivatives [207, 208], as well as hexagonal boron-nitride [209], MXenes are competitively leveraged in gas sensing applications by virtue of their outstanding electrical and optical characteristics and diverse surface functionality [210]. As gas recognition principally entails charge transfer processes between the absorbate and absorbent, MXenes’ remarkable electrical conductivity, which can be further elevated using conductive polymers as in MPCs, renders them as a potent sensing tool to recognize VOCs and non-VOCs [211].

**Fig. 13:**
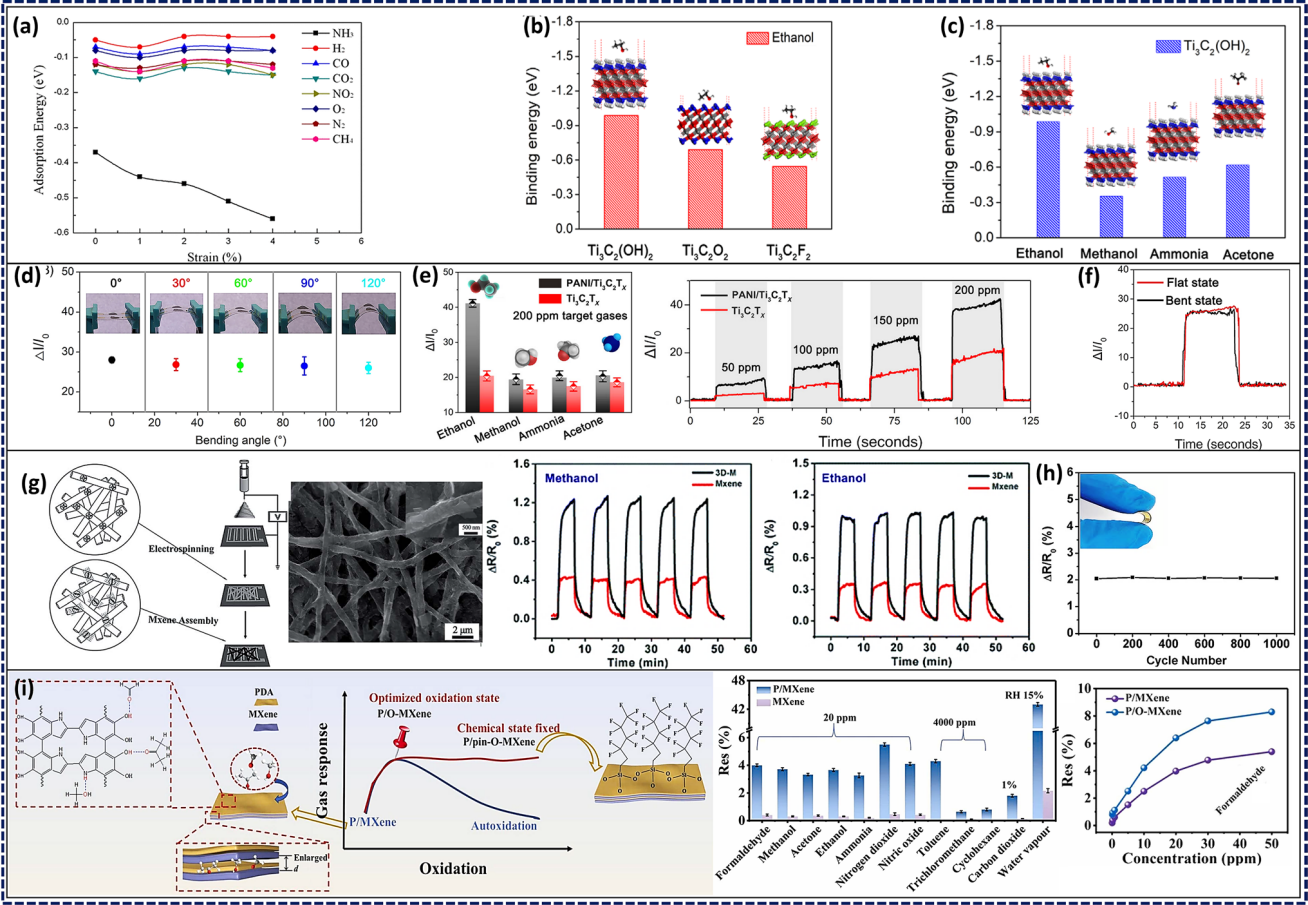
**a** Simulated adsorption energy of eight different gas molecules on Ti_2_CO_2_ monolayers as a function of applied biaxial strains, and side- as well as top-view of schematic of the pertinent adsorption. Reproduced with permission from Ref. [212], Copyright American Chemical Society 2015. **b** DFT adsorption energy values of ethanol molecule on Ti_3_C_2_(OH)_2_, Ti_3_C_2_O_2_, and Ti_3_C_2_F_2_ and **c** Comparison of adsorption energy values for ethanol, methanol, ammonia, and acetone gas molecules on Ti_3_C_2_(OH)_2_; **d** Optical images of flexible PANI/Ti_3_C_2_T_x_ sensors with the sensitivity values against varied bending angles (exposed to 150 ppm ethanol gas molecules); **e** The juxtaposition of the pristine Ti_3_C_2_T_x_ and PANI/Ti_3_C_2_T_x_ MPC in terms of selectivity for various gas targets (200 ppm) and dynamic response/recovery trends ranging within 50–200 ppm of ethanol, both performed at RT, and **f** bending stability of flexible sensors to 150 ppm ethanol gas molecules. Reproduced with the permission from Ref. [219], Copyright Wiley 2019. **g** Schematic illustration of the fabrication process, SEM image of the 3D PVA/PI/Ti_3_C_2_T_x_, as well as the resistance changes of the 3D MPC sensor and MXene sensor during 5 successive cycles of exposure to 5 ppm methanol and ethanol; **h** Sensing performance of the 3D MPC sensor at 20 ppm acetone undergoing different bending cycles. Reproduced with the permission from Ref. [220], Copyright Royal Society of Chemistry 2018. **i** Schematic illustration of P/MXene chemical structure and the pinning strategy to acquire long-term sensing stability, comparison of the sensing responses of P/MXene and MXene at 20 ppm of formaldehyde, response intensities of P/MXene and MXene to various gas species, and sensing responses of P/O-MXene and P/pin-O-MXene to various gas species (P/MXene, P/O-MXene, and P/pin-O-MXene stand for PDA/MXene, partially oxidized P/MXene, and final pinned P/O-MXene, respectively). Reproduced with the permission from Ref. [222], Copyright Wiley 2020

Prior to delving into the significance of MPCs in sensing gaseous targets with respect to their remarkable sensitivity, selectivity, and rapid response time, computational simulations have been invariably lending hand to experimental explorations as intriguing predictive tools to corroborate the MXenes’ efficacy. Density functional theory (DFT) and molecular dynamics (MD) simulations are frequently utilized from the outset of a given project as first-principles studies to envisage the viability and the optimized parameters in developing a novel MPC. Firstly, significantly higher sensitivity and selectivity of Ti_2_CO_2_ monolayer towards ammonia compared to other compounds was substantiated by Yu et al. [212] through DFT computations (Fig. 13a). Notably, the lower adsorption energy accounts for more potent affinity of materials towards each other, which, in turn, induces more favorable adsorption processes. Similarly, Xiao et al. demonstrated the competence of O-functionalized M_2_CO_2_ MXenes, where M can be either Zr, Ti, Sc, or Hf, in efficient reversible release and capture of gaseous NH_3_ [213].

MPCs provide unique heterointerface impacts between MXenes and the active polymeric materials culminating in the promotion of interfacial charge transfer, addition of extra synergetic phenomena, and heightened efficient contact with gaseous analyte for the presence of plentiful surface functionalities. In this vein, designing pliable and durable MPCs is crucial for advancing the development of high-performance wearable, real-time breath-based sensors.

#### Alcohol and Ketone Sensing

The existence of alcoholic compounds (AC), classified as VOCs, in the exhaled breath is closely entangled with variety of potential diseases like diabetes, respiratory infections, asthma, cirrhosis, and cystic fibrosis [214–217]. Ketones like acetone are indicative of diabetes when exceeding normal ranges (300–900 ppb) and reaching up to 1800 ppb in human breath [218].

Ti_3_C_2_T_*x*_ is the most explored MXenes, and its properties can be tailored by altering the surface terminal groups. While terminated with oxygen (–O) groups, Ti_3_C_2_T_*x*_ exhibits characteristics akin to a small band gap semiconductor, a metal-like character with an excellent electrical conductivity is predominant for hydroxyl (–OH), displaying remarkable electrical conductivity. This renders Ti_3_C_2_(OH)_2_ highly desirable for sensor applications operating at room temperature (RT). The –OH groups are polar and in theory known to adsorb polar analytes with similar –OH groups in gas or liquid states, which makes Ti_3_C_2_(OH)_2_ suitable for alcohol sensing. Zhao et al. [219] demonstrated through DFT calculations that the robustness of the ethanol adsorption on Ti_3_C_2_(OH)_2_ far surpasses that of Ti_3_C_2_O_2_ and Ti_3_C_2_F_2_ due to the more negative binding energies (Fig. 13b). Furthermore, the selective ethanol sensing of Ti_3_C_2_(OH)_2_ was verified by first-principle simulations (Fig. 13c). Analogous to the prior theoretical outcomes, MPCs comprised of flexible PANI/Ti_3_C_2_T_x_ nanocomposites were synthesized by an in-situ, low temperature polymerization with an attempt to preserve both Ti_3_C_2_T_*x*_ and PANI structural quality. Figure 13d manifests the high elasticity of the resultant MPCs, in which the ethanol sensitivity at a bending angle of approximately 120° was well maintained, compared to the flat state (27.4% to 150 ppm of ethanol gas molecules). Furthermore, Fig. 13e confirms that the obtained ethanol sensitivity transcends that of other gaseous target molecules and the value assigned to the MPC is 2.3 times higher than pure MXenes due to the overabundance of functional groups. These outcomes underline the pivotal role of polymer integration in enhancing MXenes’ sensor performance due to the synergistic aspects. Besides, PANI/Ti_3_C_2_T_*x*_MPCs displayed functionality in ethanol recognition at RT with rapid response/recovery time (less than 1 s for both flat and curved films) (Fig. 13f), which are essential parameters for developing real-time breath-based biosensors.

Nevertheless, superficial –OH groups of MXenes are also capable of forming potent hydrogen bonds with oxygen atoms in acetone and AC chemical structures. Accordingly, Yuan et al. [220] fabricated a 3D framework of poly(vinyl alcohol)/polyetherimide (PVA/PI) nanofibers using electrospinning on which negatively charged Ti_3_C_2_T_*x*_ was self-assembled owing to electrostatic interactions. The fabricated 3D structure provided a remarkable interconnected porous media, streamlining the access and diffusion of acetone, ethanol, and methanol molecules (Fig. 13g). PVA/PI/Ti_3_C_2_T_*x*_nanofibers sensitivity towards methanol, ethanol, and acetone was improved by virtually two-fold compared to pristine Ti_3_C_2_T_*x*_. The flexibility and reliability of the sensor were confirmed (Fig. 13h), where no significant deviation in the sensing response was observed even after 1000 bending/unbending cycles. LOD of 50 ppb and sensitivity of 0.1–0.17 ppm^−1^ with less than two-minute response and recovery times rendered these MPCs desirable in AC-acetone wearable sensors with proper functionality at RT.

The gas sensing performance of MPCs can be further enhanced through creating metal–semiconductor interfaces. Notwithstanding the metallic nature of MXenes, they can exhibit semiconducting behaviours through oxidation, particularly in case of Ti_3_C_2_T_*x*_, where the presence of terminal TiO_2_ can induce this effect [221]. Consequently, an extra-sensing mechanism known as Schottky barrier modulation can be introduced to the prior charge transfer sensing mechanism. Inspired by the mentioned phenomenon, Zhang et al. [222] took advantage of the enhanced gas sensing of MPCs with metallic-semiconducting interfaces to further underline the decisive impact of MXenes’ terminal groups on promoting VOC adsorption. They proposed a novel sensing platform consisting of Ti_3_C_2_T_*x*_decorated with PDA in interlayer spaces, which was synthesized via in-situ polymerization. The as-fabricated platform was highly sensitive and effective in detecting formaldehyde, acetone, and methanol biomarkers (Fig. 13i). PDA endowed Ti_3_C_2_T_*x*_with substantial active –NH_2_ and –OH sites, amplifying the gas response over 16-fold compared to pristine MXenes. Moreover, the optimized oxidation state was achieved at RT and retained by silane surface modification to engender a hydrophobic surface, which also inhibits further oxidation. Using this pinning strategy, gas response intensity was discernibly increased by virtue of the heterojunction properties at the interface of the gas molecules and the final MPC. Despite not being practical as elastomeric wearable sensors at this stage, this study sheds light on both the indispensable role of polymeric constituents in introducing numerous interaction sites and feasibly synthesizing MXenes with desired ratio of functional groups to fine-tune sensitivity and gas response.

#### Ammonia Sensing

Being another essential biomarker in medical diagnosis applications, ammonia (NH_3_) level monitoring has been long of interest to ensure the healthy function of the liver and kidneys. At any rate, inordinate amounts of ammonia are converted into a less noxious substance like urea in liver cells concomitant with being filtered out while passing through glomerulus to maintain the safe pH balance in the human body. Any sort of anomaly in these organs ends up with elevated levels of ammonia, especially being detectable in the mouth breath as a non-invasive approach. Chronic kidney disease (CKD) and liver dysfunction can be ascribed with high certitude to the escalated ranges of NH_3_ (1392–3660 ppb and 820–14,700 ppb, respectively) in the exhaled breath [223, 224]. Conversely, ammonia levels decline significantly in patients with obstructive lung diseases (e.g., asthma), measured to be within 11–53 ppb [225]. *Helicobacter pylori* bacteria-induced stomach infections also tends to increase the ammonia levels in breath up to 400 ppb [226].

In a pioneering study, the integration of Ti_3_C_2_T_*x*_ onto flexible PI films through solution-casting corroborated MXenes’ capability in sensing ammonia [227]. The resultant films were exploited at RT, a key parameter in exhaled VOCs sensing, with 0.21 ppm^−1^ average gas response and LOD of less than 9.27 ppm towards NH_3_. The former LOD, however, is not entirely apt for ammonia sensing in early detection applications since the value merely tackles the increased stages of CKD and liver disorders.

**Fig. 14:**
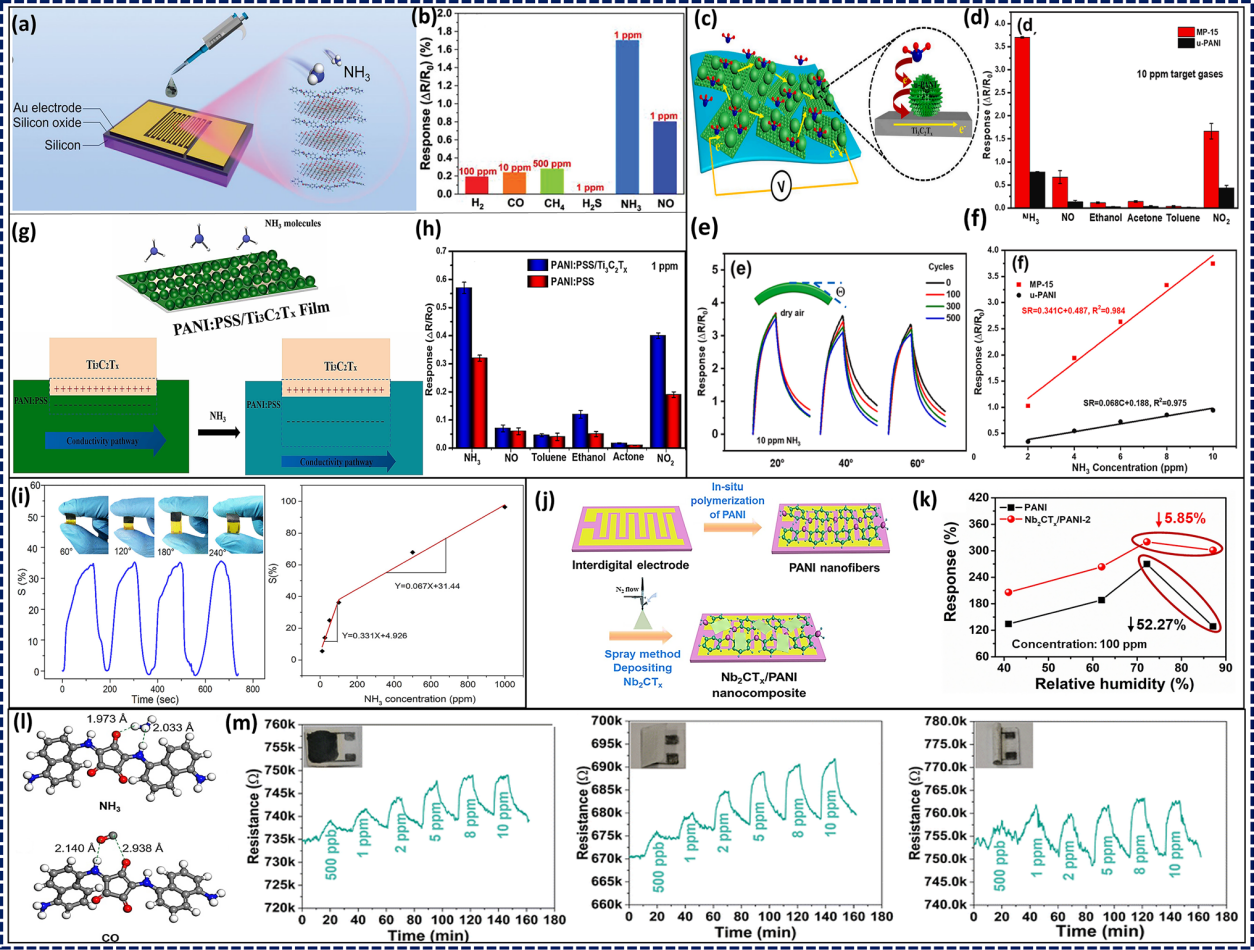
**a** Schematic of drop-castedTi_3_C_2_T_x_/PDDS hybrid films on the electrodes and **b** the selectivity of the fabricated sensor. Reproduced with the permission from Ref. [228], Copyright Wiley 2022. **c** Illustration of ammonia sensing mechanism of flexible Ti_3_C_2_T_x_/urchin-like PANI hollow nanosphere composite sensors; **d** Comparison between pristine u-PANI and MP-15 (MPC with 15 wt% aniline) selectivity to different targets; **e** Dynamic response-recovery curves under successive bending cycles with different bending angles (inset depicts the bending orientation), and **f** Response-concentration fitting plots ranging within 2–10 ppm. Reproduced with the permission from Ref. [229], Copyright Elsevier 2022. **g** Sensing mechanism of Ti_3_C_2_T_x_/PANI:PSS MPC, and **h** the comparison between MPC and pure PANI:PSS in terms of selectivity. Reproduced with the permission from Ref. [230], Copyright Elsevier 2023. **i** Gas response of the Ti_3_C_2_T_x_/PANI:PSS composite-based sensor towards 100 ppm NH_3_ at varied bending angles and the linear dependence between the gas response and ammonia concentration. Reproduced with the permission from Ref. [231], Copyright American Chemical Society 2020. **j** Fabrication process of the Nb_2_CT_x_/PANI sensors; and **k** humidity influence on the sensing response for 100 ppm ammonia. Reproduced with the permission from Ref. [232], Copyright Elsevier 2021. **l** DFT-simulated adsorption modes and adsorption distance between PDAC and two different target molecules, namely NH_3_ and CO, and **m** real-time resistance of the developed Ti_3_C_2_T_x_@PDAC paper-based sensor versus time with respect to the ammonia concentration in flat, folded, and rolled states. Reproduced with the permission from Ref. [235], Copyright Royal Society of Chemistry 2023

Incontrovertibly, MPCs possessed superior NH_3_ sensing competency. As a proof-of-concept, Zhou et al. [228] achieved a remarkably sensitive MPCs composed of Ti_3_C_2_T_*x*_intercalated with poly-(1,4-diamino-2,5-dichlorobenzene-squarine) (PDDS) via fast microwave-assisted in-situ polymerization. Not only the MXene interlayer spacings increased due to the presence of the PDDS, which facilitated the efficient interaction with NH_3_ molecules, but also PDDS offered assorted types of active sites for forming hydrogen bonds and ion–dipole interactions with the target. Due to this synergistic effect, Ti_3_C_2_T_*x*_/PDDS MPCs exhibited an improved LoD of 500 ppb with a good selectivity towards NH_3_ (Fig. 14a, b). This LoD has been further enhanced to 30 ppb by developing Ti_3_C_2_T_*x*_/PANI nanosphere MPCs deposited onto the flexible polyethylene terephthalate (PET) films (Fig. 14c) [229]. The outstanding sensor performance at RT is directly pertinent to the both urchin-like morphology of the PANI hollow nanospheres, maximizing the accessible surface area, and heterojunction essence of the MPC, which shows 4.74-fold higher sensitivity toward NH_3_ compared to the bare urchin-like PANI. Besides, the high selectivity, retaining almost equal responses after 500 bending cycles, and good linear range of the as-prepared sensor are showcased in Fig. 14d–f. In a comparative investigation, doping PANI with poly(4-stryenesulfonate) (PSS) during in-situ polymerization resulted in the production of flexible PET-based PANI:PSS/Ti_3_C_2_T_x_ films with enhanced characteristics [230]. PSS successfully introduced innumerable -SO_3_H functional groups to the final composites leading to a more readily adsorption of NH_3_ molecules as well as an ultra-low LoD of 20 ppb with response value of 0.57–1 ppm at RT (Fig. 14g, h). Furthermore, the created Schottky junction between the interface of PANI:PSS and Ti_3_C_2_T_*x*_fostered more distinct resistance drop during NH_3_ sensing. As indicated in Fig. 14i, the depletion layer of the Schottky junction faces rise throughout the ammonia introduction resulting in the confinement of the conductivity pathway. This phenomenon accounts for the elevated sensitivity of MPCs with respect to each component alone due to the observed metallic-semiconducting behavior at the polymer/MXene interface. The promising outcomes derived from PANI:PSS/Ti_3_C_2_T_*x*_ towards NH_3_ sensing has also been authenticated previously by Jin et al. [231]. Unlike the previous study, they discretely fabricated MPCs, which were subsequently deposited onto PI substrates. Figure 14i demonstrates the high pliability and mechanical stability of the MPC films without any significant drop in gas response at different bending angles, and a good linear response with better sensitivity (i.e., 33.1%) in 10–100 ppm range of NH_3_ concentration.

Apart from being operable at RT, MPC-based sensors must also sustain a proper sensitivity in highly humid media to be practical as breath-based biosensors. Exhaled breath is recognized for having an extremely humid environment, which can result in relative humidity (RH) levels above 80%. Hitherto, preponderance of the investigated studies encounters appreciable drop in sensing gas molecules by increasing the RH. To surmount the challenge of water molecules cross-sensitivity during the gaseous biomarker sensing in a more efficient way, Nb_2_CT_*x*_ were coated on PANI-nanofibers/PI films via spray method as depicted in Fig. 14j [232]. Nb_2_CT_*x*_/PANI films exhibited not only a considerably higher selectivity towards NH_3_, but also an exceptional sensitivity of 301.31% in case of exposure to 100 ppm NH_3_ under high RHs (87.1%). This sensitivity outperformed that of pure Nb_2_CT_*x*_ (8.15%) and PANI (128.81%) sensors. Moreover, the sensitivity decease in high RHs is negligible for the developed MPCs compared to pristine PANI sensors (i.e., 8.94 times lower) (Fig. 14k). This can be directly related to the fact that hydrogen bonds initiated between Nb_2_CT_*x*_ and PANI play an essential role in taking up the active sites favorable for water molecule adsorption, which induced the same gas response in higher RHs.

In addition to RT and RH, response/recovery rate principally determines the feasibility of real-time measurements. In this regard, the incorporation of Ti_3_C_2_T_*x*_into cationic polyacrylamide (CPAM) empowers the resultant MPCs with an exceptional flexibility, roughly the same gas response at 90° bending, and fast response/recovery rate of 2.7/14.6 s to 150 ppm ammonia [233]. Furthermore, Ti_3_C_2_T_*x*_/CPAM MPCs offered long-term stability and high NH_3_ selectivity at RT, which highlights the meaningful contribution of CPAM to promote Ti_3_C_2_T_*x*_sensing properties and vice versa.

Quantum chemistry computations have substantiated the strong reliance of MXenes on the type of surface terminations to be implemented as promising NH_3_ sensors. In case of NH_3_ detection, Hajian et al. [234] showed through DFT calculations that the role of fluorine atoms in adsorbing NH_3_ molecules is subsidiary to that of oxygen. Consequently, a better charge transfer between ammonia and MXene can occur in oxygen-enriched MPCs. This synergistic effect can be theoretically investigated from the polymeric perspective [235]. An ion-in-conjugated polymer, namely poly(1,5-diaminonaphthalenecroconaine) (PDAC), was evaluated for ammonia sensing. By DFT calculations, it was verified that PDAC backbone groups, chiefly –O and –NH groups, can establish the most minimal adsorption distance with NH_3_ model gas compared to other gas molecules, like CO as depicted in Fig. 14l. Additionally, higher charge transfer values and most favorable adsorption energies were reported specifically for NH_3_. Furthermore, this promising theoretical outcome elicited the synthesis of core–shell Ti_3_C_2_T_*x*_@PDAC MPCs by in-situ polymerization, where PDAC shells enhanced MXenes’ sensing characteristic in two ways. Firstly, the entire surface coverage of MXenes with PDAC cushion them from oxidation resulting in an astounding mechanical stability over a span of at least 40 days. Secondly, PDAC served as an intermediary between MXene cores and analytes to pave the way for further electron tunnelling and charge-transfer sensing mechanism to Ti_3_C_2_T_x_ layer. Consequently, Ti_3_C_2_T_x_ @PDAC core–shell structures outperformed pristine Ti_3_C_2_T_x_ in terms of sensitivity (2.8% ppm^−1^), LoD of 50 ppb, and being applicable as pliable printed sensors that preserve comparable sensing performance in varied deformation states, as illustrated in Fig. 14m.

#### Sensing of Non-polar Gases and Other VOCs

**Fig. 15:**
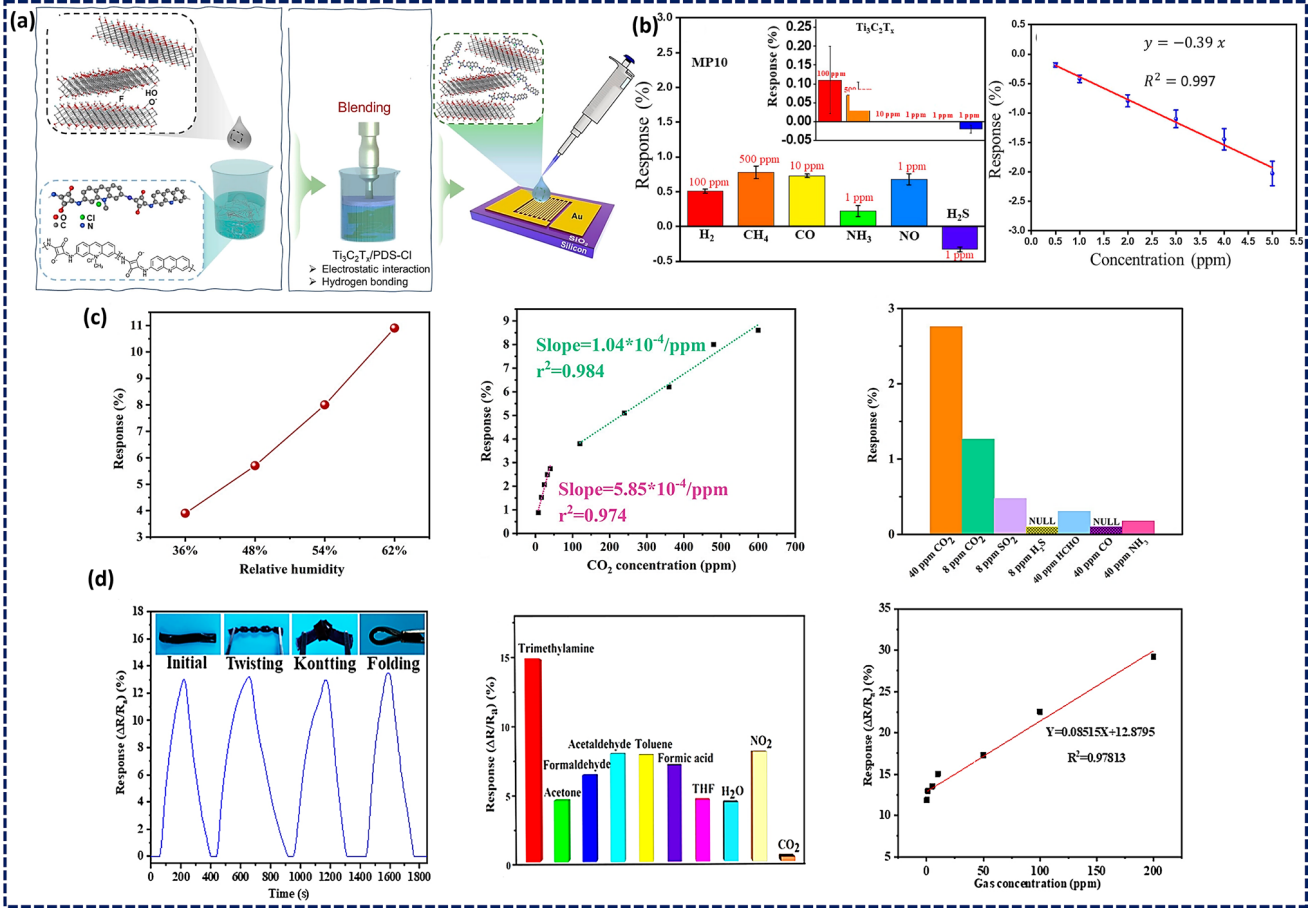
**a** Schematic illustration of Ti_3_C_2_T_*x*_/PDS-Cl nanocomposite synthesis and sensor fabrication: addition of MXene ink to the water dissolved PDS-Cl polymer, tip sonication (for 2 min at 20 W) for accelerating the physical blending, and fabrication of H_2_S sensor by drop-casting the MPCs solution on prepatterned Au electrodes; **b** Selectivity of Ti_3_C_2_T_*x*_/PDS-Cl (with 10 wt.- % of MXene: MP-10) toward 6 different analytes with a negative response for H_2_S (inset shows the selectivity of pristine Ti_3_C_2_T_*x*_), dynamic sensing response of MP-10 for various concentrations of H_2_S, and the related calibration curve. Reproduced with the permission from Ref. [237], Copyright American Chemical Society 2023. **c** Response of the ternary N-doped- Ti_3_C_2_T_*x*_/PEI/rGO as a function of RH, the pertinent linear fitting of response toward 8–600 ppm CO_2_, and selectivity under 62% RH. Reproduced with the permission from Ref. [240], Copyright American Chemical Society 2020. **d** Dynamic responses of the MXene@AuNP DN hydrogel sensor to 1 ppm TMA under multiple deformation states (initial, twisting, knotting, and folding states), selectivity of the gas sensor, and the acquired linear fitted response versus TMA concentration at RT. Reproduced with the permission from Ref. [242], Copyright American Chemical Society 2022

Even though the majority of developed MPCs are deployed as ammonia and alcohol/ketone sensors, there are still some reported sensors for the detection of other breath biomarker. With this regard, H_2_S has been established as an alternative biomarker for the early-stage diagnosis of small intestinal bacterial overgrowth (SIBO) [236]. A recent study focused on the conjugation of Ti_3_C_2_T_x_ with poly[3,6-diamino-10-methylacridinium chloride-co-3,6-diaminoacridine-squaraine] (PDS-Cl) with elevated sensing features compared to pristine MXene (Fig. 15a) [237]. The Ti_3_C_2_T_x_ /PDS-Cl nanocomposites were produced through straightforward in-situ physical blending. The resulting MPCs exhibited a singular negative response for H_2_S, which was about thirty times higher than that of Ti_3_C_2_T_x_ at 1 ppm inlet gas. The Ti_3_C_2_T_x_/PDS-Cl MPCs viability as H_2_S (Fig. 15b) sensors were further demonstrated by their low LoD (500 ppb), good repeatability, and decent stability. The authors also conducted DFT studies to elucidate the counter-intuitive negative responses resulting from H_2_S adsorption. They verified the interplay between intercalation and charge transfer mechanisms, which one can dominate over the other by considering two conditions First, the competition between gas molecules and omnipresent water molecules pertinent to the adsorption onto the MXene surface, and second, the type of MXene surface terminations. H_2_S was shown to possess a comparable adsorption energy to that of H_2_O in case of mixed-terminated MXenes (i.e., T_x_ = O_0.50_OH_0.25_F_0.25_), which precludes H_2_S molecules to be easily displaced from the surface by water. Hence, H_2_S molecules proceed with charge transfer mechanism inducing an amplification in conductivity (decreased resistance and negative response) rather than positioning between layers to elongate the interlayer space (i.e., increasing resistance and positive response).

Additionally, NO_x_ biomarkers are studied in depth as potential indicators of chronic pulmonary obstructive diseases, cystic fibrosis, and liver transplant rejection [199]. Sun et al. [238] introduced an MPC-based NO_x_ sensor by creating cobalt tetroxide (Co_3_O_4_)@polyethylenimine(PEI)/Ti_3_C_2_T_x_ nanocomposites, capable of detecting ppb-levels of NO_x_, a concentration found in the breath of people with asthma (> 50 ppb). The sensor operates at RT with an excellent selectivity, a fast response time of less than 2 s, and a low LoD of 30 ppb. Numerous defects in MXene and high surface area of Co_3_O_4_ alongside with the PEI role in facilitating the charge transfer between the two former components enabled synergistic processes, which led to remarkable NO_x_ adsorption.

Moreover, CO_2_ is deemed to be a key and widely exercised breath-borne biomarker in healthcare, with its measurement during respiration commonly referred to as capnography [239]. To detect trace levels of CO_2_ at RT, Zhou et al. [240] proposed nitrogen-doped Ti_3_C_2_T_x_/PEI MPCs, which were further adorned with rGO. The study revealed a wide range detection of 4–3000 ppm under 62% RH at RT, reasonable selectivity, and fair repeatability (Fig. 15c). The governing synergy responsible for the enhanced CO_2_ sensing stems from the ternary essence of the MPC, where conductive nitrogen-doped MXenes provided multitudinous interaction sides for CO_2_ and water co-adsorption, while rGO further enhanced the charge transfer. The basic amino groups of PI fostered reversible CO_2_ adsorption via acid–base reactions or base-catalyzed hydration in the presence of water.

Real-time monitoring of trimethylamine (TMA) in exhaled breath can also reveal the potential for end-stage renal disease [241]. Double-network (DN) hydrogel nanocomposites comprised of gold nanoparticle (AuNP)-modified Ti_3_C_2_T_x_ as primary sensing units and PAM/carboxymethyl cellulose (CMC) were synthesized by UV-induced polymerization [242]. The resultant MPCs, as demonstrated in Fig. 15d, are empowered with ultra-stretchability (313%) that retains practically the same TMA selective response under various mechanical deformations at RT. This robust tensile strength (1.79 MPa) was ascribed to the multiple hydrogen-bonding interactions in the hydrogel network.

The number of studies on non-polar gases, like H_2_, toluene and xylene, has remained scarce due to the difficulty in forming strong interactions at RT with the copious functional groups present in MPCs. Yet, Lee et al. opted for single- or few-layered V_2_CT_*x*_ deposited on PI to detect H_2_ at RT [204]. They verified a high sensitivity (24.3%) to 100 ppm of hydrogen, which can be assessed as biomarker for SIBO and intestinal glucose malabsorption [243]. Notwithstanding the lack of experimental evidence on MPCs, the incorporation of polymers in MXenes is anticipated to boost the sensing performance by virtue of introducing new functional groups. As an example, DFT simulations suggest that sulfur groups can serve as active sites for toluene recognition [244]. Alternatively, instead of applying high temperature media and meticulous processing steps to attain well-doped MXenes, polymers with –S groups might have the competitive enhancing characteristics for toluene sensing.

To put it in a nutshell, a vast majority of to-date experiments have harnessed MPCs as promising tools in detecting ammonia, alcohols, and acetone as breath biomarkers for a probable disease diagnosis. It has been corroborated that the sensing performance of MPCs outperformed that of pristine MXenes by creating a unique interface between the MXenes and the active polymeric materials. This led to an increase in charge transfer at the interface, synergistic effects, and better contact with gaseous analytes due to the presence of various surface functionalities as well as endowing MPCs with flexibility, which makes them applicable as wearable breath-based sensors.

## Development of Advanced MPC Sensors

In recent times, there has been a rise in the development of innovative van der Waals 2D nanostructured materials for future-generation applications, which exhibit comparable physicochemical properties to graphene and its derivatives, while also possessing advantageous characteristics such as hydrophilicity, high stability, ease of functionalization accessibility, larger flake size, improved yield, and superior machine processability [245–247]. Experiments have shown that incorporating polymers into MXenes or vice versa results in MPCs with better and improved characteristics [47, 248]. These properties include an exceptional conductivity and flexible mechanical characteristics, which are attributed to the interfacial multi-interactions between both phases. When MXenes are introduced into polymers, they retain their conductivity even under applied mechanical strain, making them perfect for applications demanding flexibility. The distinct features of MXenes, such as their large surface area and conductivity, make them sensitive to minor mechanical changes, allowing for precise sensing. Polymer-supported MXene composites can be engineered to induce multi-functionality. In addition to stretchability, these sensors can integrate other features, such as self-healing capabilities or environmental responsiveness, thus further expanding their potential applications. This makes them promising candidates for the development of advanced sensors in future generations. Besides this, MPCs have been anticipated for diverse purposes, including but not limited to energy production and storage, electromagnetic protection, actuation, and optical limiting. The global requirement for an intelligent control and monitoring strategies, such as gas/vapor sensors for air contaminants, has increased. Gas and vapour sensors have a wide range of applications beyond air quality monitoring, which include intelligent farming, military operations, smart cities, public safety, food security, safety at work, and therapeutic diagnosis.

Airborne NH_3_ has been identified as a biomarker for the diagnosis of renal and gastric disorders resulting from Helicobacter pylori infection, as well as COVID-19 in recent times [223, 249]. The concentration of NH_3_ in the exhaled breath of a patient with renal disorder falls within the range of approximately 0.82–14.7 ppm. Gas/vapor sensors can potentially replace conventional diagnostic techniques that are costly and time-consuming by enabling real-time detection of NH_3_ in low-trace concentration ranges [223, 249]. The proliferation of diverse consumer needs has led to the growth of the global sensor industry, which attained a global market worth of 190 billion USD in 2021. It is anticipated that this industry will expand further and reach a market value of 1 trillion USD by 2025 [250]. The sensor development necessitates the incorporation of sophisticated attributes such as fast and minimal trace detection, durability, affordable, easy manufacturing, simplified and transportable structure, which is a hot topic of on-going research. In this context, a set of PEDOT:PSS/MXene nanocomposites were synthesized using in-situ polymerization. These materials were subsequently applied onto polyimide (PI) substrates to construct NH_3_ sensors [231]. At RT, the sensor exhibited a robust gas response of 36.6% towards 100 ppm of NH_3_, with a response time of 116 s and a recovery time of 40 s. Furthermore, it was observed that the sensor displayed superior sensing capabilities compared to sensors solely composed of PEDOT:PSS or Ti_3_C_2_T_*x*_, which implies a synergistic effect between PEDOT:PSS polymers and Ti_3_C_2_T_*x*_. Wang et al. proposed a novel approach for improving the NH_3_-sensing response, which involves the use of a selective sensor composed of PANI nanofibers-supported Nb_2_CT_*x*_ (Nb_2_CT_*x*_/PANI) that is directly driven by triboelectric nanogenerator (TENG) [232]. The results indicated a noticeable improvement in both the NH_3_-sensing response and response time of the Nb_2_CT_*x*_/PANI-2 sensor. This suggests that the sensor exhibits a strong linear response across a broad sensing range of 1–100 ppm NH_3_ at RT under 87.1% RH. The study delved deeper into the mechanism behind the heightened gas sensing properties, which can be primarily assigned to the improvement effect of the p–n junction on gas sensing. Yuan et al. presented a novel approach for the fabrication of flexible and high-performance VOC sensors utilising 3D MXene frameworks (3D-M) fabricated by electrospinning [220]. The 3D structure was fabricated via the electrospinning technique utilising an aqueous solution of cationic polymer. Electrostatic interaction between negatively charged MXene sheets and the surfaces of the polymer fibres can lead to the formation of a 3D-M. This 3D structure produced highly interconnected porous structures that allow gas molecules to easily access and diffuse. The resulting sensors demonstrated a notable sensitivity (0.1–0.17 ppm^−1^), a low experimental detection limit (50 ppb), a broad sensing range (from the ppb level to a saturated vapour), excellent flexibility (no signal degradation for 1000 bending cycles), and reversibility for different VOCs, such as acetone, methanol, and ethanol, in the ppb range. These findings suggest a significant potential for the development of wearable and practical VOC sensors. Interestingly, Zeng et al. reported the prototype of MXene/PEDOT:PSS-modified flexible interdigitated electrodes in conjunction with system-level integration of CRISPR-Cas12a-mediated target-activated gas [251]. This study aimed at developing a method for the real-time and continuous identification of human papillomavirus (HPV)-related DNA using portable smartphone visual readout and producing reactions. The irregular abrasive paper stencil printing method was used to create a Ti_3_C_2_T_*x*_-PEDOT:PSS /PDMS-based piezoresistive sensor with a randomly distributed contact surface and spinous microstructures. The Ti_3_C_2_T_*x*_-PEDOT:PSS/PDMS with unique microstructure demonstrated a good force-to-electric conversion ability. To provide additional evidence of biosensing capabilities, the CRISPR-Cas12a system incorporated magnetic bead-ssDNA-Au@Pt nanoparticles (MB-ssDNA-Au@PtNPs) conjugates as a means of signal transduction, thereby substituting the typical fluorescent and quencher-labeled ssDNA reporter. Upon binding to the target HPV-related DNA, the Cas12a-crRNA duplex complex triggers the activation of the indiscriminate ssDNA cleavage activity of Cas12a, leading to the degradation of the ssDNA in MB-ssDNA-Au@PtNPs and the subsequent release of Au@PtNPs. The released and separated Au@PtNPs catalyse the conversion of H_2_O_2_ to O_2_, and the generated O_2_ compresses the Ti_3_C_2_T_*x*_-PEDOT:PSS/PDMS piezoresistive device in a 3D-printed home-made pressure-tight vessel, causing the current to increase. The Ti_3_C_2_T_*x*_-PEDOT:PSS/PDMS piezoresistive device was coupled to a wireless system through Bluetooth integrated circuit to provide real-time visualisation of experimental outcomes as well as data storage, processing, and transmission features.

## Challenges, Potential Remedies, and Future Strategies

**Fig. 16:**
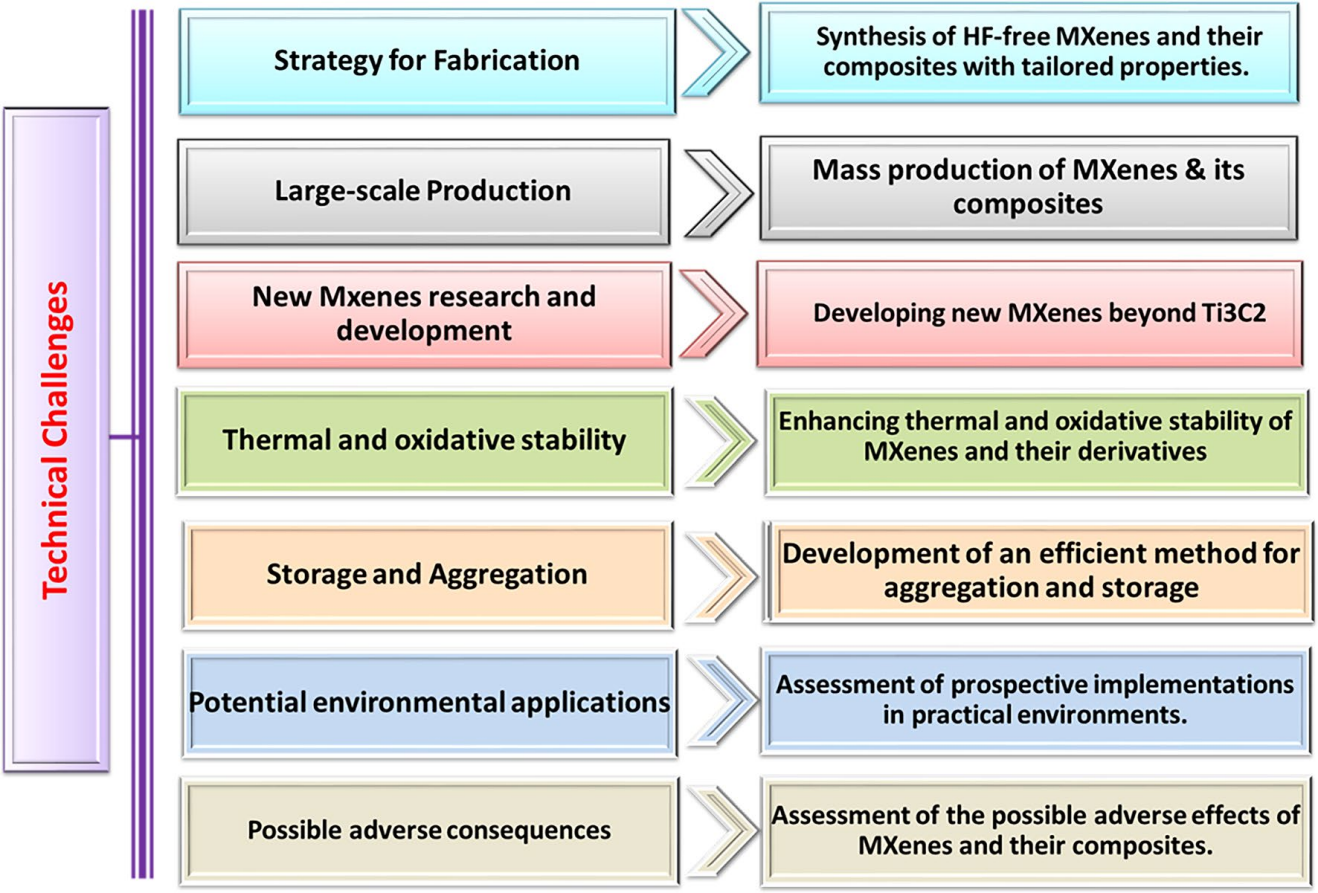
Significant technical obstacles in the application of MXenes and their composites

The potential applications of MXenes are significant; however, there exist several technical challenges that must be addressed to fully realise the benefits of these nanomaterials, as illustrated in Fig. 16. To overcome these obstacles, the following research avenues are recommended for prospective enhancement:

- One potential avenue for further research involves exploring alternative etching agents to corrosive HF, such as difluoride salts NH_4_HF_2_, or sodium and potassium bifluoride salts (NaHF_2_, and KHF_2_), as well as investigating the hydrothermal procedure. The implementation of such substitutions has the potential to enhance adsorption capabilities through the provision of surfaces that are rich in oxygen. The utilisation of environmentally sustainable chemicals or fluoride-free methods in the synthesis of MXenes and their composites, along with the exploration of alternative MAX phases, represents a potentially fruitful avenue for further investigation in this field.
- One additional difficulty pertains to the development of cost-efficient methodologies for the large-scale manufacturing of MXenes and their corresponding composites. This will expand their utilization across diverse domains and facilitate a substantial advancement toward commercialization.
- The performance of MXenes is notably compromised due to their susceptibility to oxidation in ambient conditions. Further research is required to enhance the chemical stability of composites, thereby augmenting their utility.
- The incorporation of magnetic characteristics or the addition of MXenes to gel or beads has the potential to enhance the efficacy of handling and removal processes.
- The consideration of morphological aspect in hybrid architectures holds significant importance. According to reports, the morphology of MXenes is characterised by accordion-like multilayers rather than 2D nanosheets, thereby limiting their applicability in various domains. The surface area available for electrolyte infiltration is greater in delaminated MXenes as compared to multilayer MXenes. One potential challenge that must be addressed in electrical applications pertains to the reduction in conductivity that occurs with an increase in the number of layers.
- One additional obstacle in the utilization of MXenes and their composites pertains to the aggregation and storage of these materials, necessitating further investigation to optimize their efficacy. The preservation of MXenes dispersions through sub-zero storage represents a potential area of investigation for future research.
- Another issue is determining the life cycle of MXenes and their negative effects on the ecosystem because they can be absorbed through the skin, GI tract, and respiratory systems. Despite the limited availability of data on the toxicological effects of MXenes, recent studies have raised concerns regarding the potential adverse impacts of these materials on aquatic ecosystems and human health.

## Summary and Outlook

MXenes-based elastomer mimetic sensors are a new class of flexible sensors that have received a lot of attention because of their unusual combination of qualities like high sensitivity, a wide strain sensing range, and significant mechanical properties. The present review encompasses an overview of the principal findings from recent literature of MXenes-based elastomer mimetic nanocomposites and their underlying the impact for the use of flexible sensors. Herein, we have discussed different types of MXenes-based stretchable sensors based on soft matrix, biopolymer, rubber, and textile. Also, breath-based biomarker and some advanced sensors have been discussed. Overall, the synthesized research suggests that MXenes possess exceptional characteristics that result in significant enhancements across all properties of polymeric composites, even when present in small quantities. The proper dispersion and enhancement of mechanical properties of polymers were attributed to the presence of functional groups on the structure of MXenes and the formation of H-bonding. These sensors have exhibited significant potential for diverse applications, including body motion detection, electronic skins, wearable devices, and medical diagnostics. For example, they have been employed for the purpose of tracking physical motions, identifying muscular contractions, and quantifying physiological metrics such as heart rate and respiration rate. Furthermore, they have been utilized in the field of soft robotics, wherein they are incorporated with actuators to establish sophisticated systems that imitate human motions. Future investigations are expected to encompass the advancement of MXene in polymeric composites, including but not limited to V_2_CT_x_, Mo_2_C, V_2_C, Nb_2_C, and V_4_C_3_. Additionally, analytical modelling of MXene/polymer composites, more sophisticated designs, integration with other technologies, and practical applications are anticipated to be explored.

The commercialization potential of MXene composite sensors is an intriguing topic in the realm of materials science and sensor technology as they move from sterile laboratory settings to real-world applications. These sensors have shown an impressive sensitivity and selectivity in lab settings, but there are still significant obstacles to overcome before they can reach their full potential and be widely integrated into the market. The inability for up-scaling is a major challenge. Laboratory-scale synthesis processes may not be immediately transferable to large-scale manufacturing, thus necessitating the creation of durable and efficient production systems that can fulfil the expected market demand. This requires not just fine-tuning synthesis techniques but also making sure each MXene batch is of the same high quality. Moreover, financial aspects and budget are also crucial since the marketability of MXene sensors depends on their low price and ability to compete. There is a need to develop cost-effective production pathways since research-focused synthesis may be too expensive for a wider implementation. Therefore, a balance between low cost and high performance must be found, which requires more applied research in the future.

Additionally, the durability and long-term performance of MXene-based sensors are critical. For sensors to be useful in the real world, they need to maintain their performance and dependability over time. MXenes have exhibited vulnerability to environmental variables such as relative humidity and oxygen exposure, which requires the development of protective coatings or encapsulating strategies to enhance their endurance. Regulation conformity is an additional aspect to consider. Important for the successful commercialization is the conformity with applicable safety, health, and environmental impact norms and laws. To guarantee that MXene sensors follow all applicable standards, it is necessary to put them through extensive testing and certification procedures. Another difficulty relates to the precise tailoring of the sensors' sensitivity, reaction time, and selectivity. The development of MXene composites that may be adapted to the specific needs of various markets and applications is an ongoing process. In conclusion, due to their exceptional characteristics and adaptability, sensors based on MXene composites have considerable economic potential. Scalability, costs, long-term stability, regulatory compliance, and application-specific modification are just few of the hurdles that must be overcome before these sensors may be used outside of the lab. The transformational promise of MXene-based sensors in fields as diverse as environmental monitoring, healthcare, and beyond will only be fully realised if these obstacles can be overcome.
